# Breaking down silo thinking: the role of perceived supervisor support, psychological safety, and staff engagement in Ghana’s healthcare sector

**DOI:** 10.3389/fpsyg.2026.1921650

**Published:** 2026-10-20

**Authors:** Hayford Asare Obeng, Richard Arhinful, Leviticus Mensah, Abdullah Sarwar, Tarik Atan

**Affiliations:** 1 Department of Business Administration, World Peace University, Nicosia, Cyprus; 2 Department of Business Administration, Onbes Kasim Kibris University, Nicosia, Cyprus; 3 Symbiosis Centre for Management Studies, Symbiosis International (Deemed University), Bengaluru, Karnataka, India; 4 Faculty of Management, Multimedia University, Cyberjaya, Malaysia; 5 Department of Business Administration, Cyprus International University, Nicosia, Cyprus

**Keywords:** leadership, perceived supervisor support, psychological safety, silo thinking, staff engagement

## Abstract

**Introduction:**

This study examines the associations between silo thinking and staff engagement by considering the mediating role of psychological safety and the moderating role of perceived supervisor support, drawing on Social Capital Theory and Social Exchange Theory.

**Methods:**

A purposive, multi-stage sampling approach was used to collect data from 363 healthcare professionals working in public and private hospitals in Ghana. Data were analysed using partial least squares structural equation modelling (PLS-SEM). Harman’s single-factor test and the full-collinearity test were employed to assess common method bias.

**Results:**

The findings indicate that silo thinking was significantly and negatively associated with both staff engagement and psychological safety. Furthermore, psychological safety was significantly and positively associated with staff engagement and partially mediated the relationship between silo thinking and staff engagement. Additionally, perceived supervisor support significantly moderated the relationships between silo thinking and staff engagement and between silo thinking and psychological safety; however, it did not significantly moderate the relationship between psychological safety and staff engagement.

**Conclusion:**

The findings extend the complementary perspectives of Social Capital Theory and Social Exchange Theory by highlighting the importance of organisational relationships and supportive supervisory practices in understanding staff engagement within Ghana’s public and private healthcare sectors. Given the study’s focus on healthcare professionals in Ghana, future research could employ longitudinal designs and examine these relationships across different healthcare systems and cultural contexts.

## 1 Introduction

Staff engagement significantly affects organisational efficiency, especially in high-pressure service sectors like healthcare, where committed employees are more likely to deliver safe, high-quality patient care and maintain performance under pressure (Bakker and Albrecht, 2018; Albrecht et al., 2021). Healthcare engagement is inherently relational, shaped by the quality of interpersonal relationships, trust, and cooperation across professional fields, as well as other key factors (Jeske and Olson, 2025; Asare Obeng and Atan, 2025). Szilvassy and Širok (2022) argue that staff engagement in healthcare organisations is shaped by the quality of interactions, support, and trust between employees and their superiors. Despite its recognised significance, studies repeatedly indicate that most healthcare organisations struggle to maintain effective staff engagement, particularly in complex healthcare environments characterised by high workloads, unclear professional boundaries, and fragmented coordination (Ominyi et al., 2025; Atan and Obeng, 2024). Among the organisational conditions that may contribute to this challenge, silo thinking has received comparatively limited empirical attention despite its potential to undermine collaboration, trust, and staff engagement in healthcare settings.

Silo thinking is an important but often overlooked source of fragmentation. It is a structural–relational condition in which departments or professional factions function independently, obstructing communication between divisions and cooperation towards a common goal (Popęda, 2025). In healthcare settings, where collaboration is crucial for effective service provision, silo thinking undermines interdisciplinary connections, limits information sharing, and erodes confidence among professional groups (Jeske and Olson, 2025). Consequently, employees may have fewer opportunities for collaboration and support, reducing the relational resources essential to maintaining full engagement in their tasks (Qin, 2025). This fragmentation obstructs the formation of shared standards and reciprocity, which are essential for maintaining effective working relationships. From an organisational standpoint, silo thinking is a structural phenomenon rather than an individual psychological experience, so it should be distinguished conceptually from employees’ views on psychological safety and supervisory support (Hwang et al., 2026). This research focuses on the individual employee perspective and examines how employees’ views on silo thinking within their organisations relate to their work engagement. Understanding this relationship is particularly important in resource-constrained healthcare systems such as Ghana’s, where structural disintegration may intensify relational disruptions and disengagement (Yesuf, 2025).

Prior research on staff engagement has concentrated on individual resources (such as resilience and motivation) and established organisational practices, encompassing leadership approaches and human resource frameworks (Albrecht et al., 2021). However, the mechanisms through which structural barriers, including silo thinking, influence engagement remain insufficiently examined. The influence of silo thinking on staff’s psychological safety, defined as the shared belief that individuals can express their views, ask questions, or acknowledge mistakes without the fear of negative consequences on their relationships, has been inadequately explored (Edmondson, 1999). In contrast to silo thinking, a structural issue within an organisation, psychological safety reflects an employee’s perception of the interpersonal work environment as safe for taking interpersonal risks. Psychological safety is inherently relational, emerging from continuous interpersonal interactions and demonstrations of respect, trust, and support (Ip et al., 2025). Barriers that obstruct collaboration and communication among employees might foster an unsafe workplace, leading to reduced staff engagement (Jones et al., 2024; Obeng and Atan, 2024). When staff believe that voicing concerns or requesting help could lead to adverse social repercussions, they are less inclined to engage actively, share information, or fully commit to their job responsibilities. Thus, psychological safety serves as a crucial mechanism through which organisational fragmentation can reduce staff engagement. Accordingly, psychological safety may mediate the relationship between silo thinking and staff engagement.

Furthermore, the relational role of supervisors has not been distinctly articulated in relation to the silo thinking–engagement dynamic. Perceived supervisor support is acknowledged as a crucial asset for staff wellbeing and engagement. However, it is often examined as a direct precursor rather than a relational element that shapes employees’ management of institutional challenges (Helaß et al., 2025). In contrast to psychological safety, which denotes employees’ views on the relational climate in their workplace, perceived supervisor support specifically assesses employees’ perceptions of the extent to which their direct supervisors appreciate their input, prioritise their wellbeing (Mikkelson et al., 2026), and offer continuous professional support (El Mountasser and Sahraoui, 2025). This study defines supervisor support as a relational resource that reflects employees’ perceptions of caring, accessibility, and encouragement from their immediate supervisors, rather than as formal supervision, coaching, or mentoring structures (Rice et al., 2025). This distinction matters because not every healthcare worker has the opportunity for organised developmental engagements; nonetheless, perceived support from supervisors remains a universally relevant, daily relational occurrence (Chan et al., 2025). Supportive supervisors can help employees manage the relational challenges posed by silo thinking by fostering trust, enhancing communication, and motivating staff to remain engaged despite organisational barriers (O’Connell, 2026). Such positive relationships may also increase employees’ likelihood of reciprocating positive treatment by strengthening commitment and engagement in their job responsibilities.

We define perceived supervisor support as a boundary condition that functions across various relationship trajectories (Iser-Potempa et al., 2026). Supervisor support is expected to moderate the negative effects of silo thinking on psychological safety, help reduce the direct negative impacts of silo thinking on staff engagement, and strengthen the positive association between psychological safety and engagement. Supervisor support stabilises relationships, reinforces them, and promotes staff commitment to their tasks (Talukder, 2019).

This research utilises both Social Capital Theory (Coleman, 1988; Nahapiet and Ghoshal, 1998) and Social Exchange Theory (Blau, 1964) to explain these relationships. Social Capital Theory serves as the main theoretical framework for understanding how relational structures affect employee outcomes. In contrast, Social Exchange Theory offers a complementary perspective on employees’ behavioural reactions to working relationships. The next section discusses how these theoretical viewpoints support the proposed hypotheses.

Therefore, this study draws on these complementary theoretical perspectives to examine the relationship between silo thinking and staff engagement, considering the mediating role of psychological safety and the moderating role of perceived supervisor support in the Ghanaian healthcare sector. This study seeks to offer answers to the following questions:

1. What is the effect of silo thinking on staff engagement and psychological safety?
2. What is the effect of psychological safety on staff engagement?
3. To what extent does psychological safety mediate the relationship between silo thinking and staff engagement?
4. How does perceived supervisor support moderate the relationships between silo thinking and staff engagement, silo thinking and psychological safety, and psychological safety and staff engagement?

This study presents three notable contributions:

1. This research advances understanding of staff engagement by recognising silo thinking as a structural–relational impediment rather than focusing exclusively on person- or HR-related elements.
2. It expands Social Capital Theory by using empirical data to show how relational mechanisms such as psychological safety and perceived supervisor support can mitigate the impact of silo thinking on engagement.
3. The study offers contextual insights from a growing healthcare system characterised by significant relational conflict and structural fragmentation, utilising data from public and private organisations in Ghana. The study explains how structural fragmentation affects participation and emphasises the importance of supervisor support as a vital balancing factor in complex healthcare settings.

Together, these contributions provide a relationally based explanation of the association between silo thinking and staff engagement in healthcare environments.

## 2 Theoretical background and hypothesis development

### 2.1 Theoretical foundation

This research draws on Social Capital Theory, which posits that social networks, trust, and shared norms create value by enabling coordinated efforts and improving individual and organisational outcomes (Ge et al., 2025; Pillai et al., 2017). In challenging professional environments like healthcare, social capital creates a relational foundation that facilitates knowledge sharing, fosters trust, and maintains collaborative behaviour across professional boundaries (Shafaei et al., 2024; Aditchere and Angmortey, 2025). This research incorporates Social Exchange Theory (Blau, 1964) to offer a more comprehensive understanding of employee behaviour, suggesting that employees respond to positive work-related relationships with affirmative attitudes and actions. Social Capital Theory serves as the principal theoretical framework by explaining how workplace relationships and relational resources are formed. At the same time, Social Exchange Theory builds on this view by explaining employees’ behavioural responses to those relationships.

Social capital consists of three elements: structural, relational, and cognitive. Structural capital refers to the organisation of relationships among individuals, including both formal and informal networks that enable interaction (Claridge, 2018). Relational capital denotes the quality of relationships based on trust, reciprocity, and mutual respect (Abd-Elrahman and Ahmed Kamal, 2022; Chowdhury et al., 2025). Cognitive capital includes shared knowledge and interpretations that promote cooperation among staff (Zhang, 2026). Together, these factors create an environment that promotes employees’ psychological safety and engagement at work.

Silo thinking is a systemic problem for social capital in this regard. Silo thinking reduces structural relationships and shared cognitive frameworks by isolating departments and obstructing cross-boundary collaboration (Jones et al., 2024). This fragmentation reduces relational capital by limiting opportunities for trust and reciprocity, leaving employees feeling less connected and engaged (Panteli et al., 2020). In healthcare organisations, where teamwork is crucial for delivering optimal patient care, interpersonal conflicts can significantly influence staff engagement and collaboration (Pattnaik and Sahoo, 2021).

Psychological safety may be regarded as a relational manifestation of social capital. Staff who perceive substantial relational capital, characterised by trust and favourable encounters, are more predisposed to articulate their views, raise issues, and offer suggestions without fear of negative consequences (Ip et al., 2025; Hallam et al., 2023). Psychological safety, in contrast to silo thinking, concerns employees’ views of workplace interpersonal dynamics rather than the organisation’s structural attributes. Conversely, when silo thinking limits structural and relational capital, psychological safety may decline, negatively impacting the relational channels that foster engagement (Campos et al., 2025). From the standpoint of Social Exchange Theory, psychologically safe environments foster employees’ reciprocation of supportive workplace relationships through enhanced engagement (Tan et al., 2025).

Perceived supervisor support serves as a vital relational reinforcement mechanism within this framework. This research reconceptualises supervisor support not merely as a direct antecedent to engagement but as a moderating factor that influences the intensity of the relational pathway (Wang and Fu, 2024). Supportive supervisors may minimise structural fragmentation by fostering relational capital across teams, bolstering trust, clarifying shared meanings, and demonstrating inclusive engagement strategies (Joo et al., 2016; Qureshi, 2024). Consistent with Social Exchange Theory, employees who recognise supportive supervisory relationships tend to reciprocate this support by demonstrating stronger commitment and engagement (Rajâa and Mekkaoui, 2025). Supervisor support is expected to reduce the negative effects of silo thinking on psychological safety, diminish the negative repercussions of silo thinking on engagement, and strengthen the positive relationship between psychological safety and staff engagement (Elshaer et al., 2025; Singh et al., 2018). In this context, the supervisor acts as a relational stabiliser, preserving or reinforcing social capital during structural conflict.

In summary, Social Capital Theory provides the main framework for explaining how organisational ties shape access to relational resources. In contrast, Social Exchange Theory complements this viewpoint by explaining employee reactions to these relational encounters. Collectively, these ideas establish the theoretical basis for investigating the associations between silo thinking, psychological safety, perceived supervisor support, and staff engagement within healthcare organisations.

### 2.2 Hypotheses development

#### 2.2.1 Relationship between Silo thinking and staff engagement

Silo thinking undermines organisational effectiveness by causing departments and professional groups to function in isolation, thereby obstructing information sharing, cooperation, and interdepartmental communication (Jeske and Olson, 2025). Social Capital Theory posits that silo thinking erodes both structural and cognitive capital (Jones et al., 2024). Broken networks obstruct cross-boundary communication and reduce collective understanding. This fragmentation hinders communication and understanding among individuals, thereby reducing staff engagement (Chen et al., 2025).

Staff engagement is a positive psychological state linked to employment, defined by vigour, dedication, and absorption (Wijerathna and Bandara, 2023). Engagement has historically been seen as an individual motivational state, although contemporary studies increasingly acknowledge its social context and the impact of workplace relationship quality (Li et al., 2025). In healthcare settings, where interdependence, teamwork, and trust are crucial for optimal performance, engagement is maintained through continuous relational interaction that promotes significance, inclusivity, and a shared objective (Okello and Gilson, 2015).

Silo thinking weakens relational dynamics by limiting communication and obstructing collaboration (de Waal and van den Berg, 2025). Research by Musendekwa (2025) indicates that silo thinking hinders interdepartmental contact and the attainment of shared goals, leading to employee isolation and diminished contributions towards organisational objectives (Jones et al., 2024). From a social capital perspective, reduced structural connectivity erodes relational capital, reducing the trust and reciprocity that motivate employees to dedicate cognitive and emotional resources to their tasks (Zhang et al., 2026). Moreover, Social Exchange Theory posits that when employees experience fragmented workplace relationships marked by insufficient communication, reduced collaboration, and diminished organisational support, they are less inclined to respond with positive work attitudes and behaviours (Rajâa and Mekkaoui, 2025), such as increased engagement. When employees perceive their tasks as separate from the organisation’s goals, their engagement may shift from relational to transactional, reducing commitment and absorption (Foroudi and Fakhreddin, 2026).

Healthcare environments depend on coordinated relationships to ensure cooperative functioning (D'Amour et al., 2008). In settings characterised by silo thinking, declining interprofessional relationships obstruct mentorship, informal support, and cooperative problem-solving, limiting the relational energy that fosters engagement (Ababneh, 2026). In contrast, relationally integrated workplaces foster interpersonal connections and shared dedication, which are favourably associated with engagement and performance outcomes (Hamid and Awhinawhi, 2025; Horak et al., 2026).

Social Capital Theory explains how silo thinking reduces the relational resources needed for collaboration, while Social Exchange Theory clarifies why individuals show less engagement when workplace relationships deteriorate. Social Capital Theory asserts that silo thinking diminishes staff engagement by undermining the critical structural and social networks vital for trust, reciprocity, and mutual understanding. In healthcare settings characterised by considerable interdependence, such relational fragmentation is particularly prone to reducing employees’ willingness and ability to perform their tasks effectively. From the discussion above, the following hypothesis was developed:

*H1:* Silo thinking is significantly and negatively associated with staff engagement.

#### 2.2.2 Relationship between Silo thinking and psychological safety

Silo thinking is a persistent challenge that enterprises, especially healthcare organisations structured by profession, function, or department, must consistently address. Social Capital Theory argues that silo thinking reduces both structural and relational capital, as fragmented networks obstruct collaboration and trust among teams (Ahmad, 2025). Reduced social capital may contribute to silo thinking, thereby endangering psychological safety, which depends on reciprocal, trust-based relationships and interactions (Rajhi and Aljuhmani, 2026). From the standpoint of Social Exchange Theory, fragmented workplace relationships reduce opportunities for favourable interpersonal relationships (Zhang et al., 2025), making employees less inclined to expect helpful reactions when voicing concerns or sharing ideas (Kim et al., 2025).

Psychological safety refers to a shared belief that the work environment encourages interpersonal risk-taking, such as voicing opinions, asking questions, or admitting mistakes without fear of negative consequences (Edmondson, 1999; Edmondson and Bransby, 2023). Psychological safety is often examined within teams; nonetheless, it is fundamentally relational and stems from continuous interactions that foster inclusion, mutual respect, and support (Hallam et al., 2023; Kanerva et al., 2013). When employees regularly experience social connection and mutual understanding, they are more likely to see the environment as conducive to expression and collaboration (O'Donovan et al., 2021).

Silo thinking reduces interpersonal relationships by isolating specialists and hindering cross-boundary communication. Empirical evidence indicates that silo environments reduce opportunities for collaborative learning and collective problem-solving, which in turn affects trust within professional organisations (Remtulla et al., 2021). From a social capital perspective, diminished structural interconnectedness undermines relational capital, hindering a culture of reciprocity and shared standards that support psychological safety (Binyamin et al., 2018). Similarly, Social Exchange Theory suggests that when employees view professional relationships as lacking support or marked by minimal reciprocity, their inclination to take interpersonal risks diminishes, thereby lowering psychological safety (Dhir and Vallabh, 2025). This fragmentation could eventually make cautious communication and risk avoidance the norm, especially in highly hierarchical environments.

Research in healthcare settings consistently shows that trust, mutual understanding, and supportive interpersonal environments are essential for fostering psychological safety (O'Donovan and McAuliffe, 2020; Kanerva et al., 2013). By contrast, hierarchical pressures and fragmented work practices foster perceptions of voice suppression and interpersonal danger (Vogt et al., 2025). In organisations characterised by silo thinking, the absence of interdepartmental connections and relational cohesion reduces employees’ trust that voicing concerns would lead to support rather than reproach (Jeske and Olson, 2025).

In challenging healthcare environments characterised by extensive interconnectivity, reduced psychological safety extends beyond personal discomfort, resulting in widespread disengagement and limited collaboration (Hubbart, 2024). Despite growing recognition of the relational elements affecting psychological safety, empirical research explicitly linking silo thinking to psychological safety as a structural–relational antecedent is limited.

Collectively, Social Capital Theory provides insight into how silo thinking disrupts the relational structures that foster trust and collaboration. In contrast, Social Exchange Theory explains why employees who experience weakened interpersonal relationships become less willing to engage in behaviours that involve interpersonal risk. Social Capital Theory contends that silo thinking reduces psychological safety by undermining structural connections and weakening relational trust within and among professional groups (Guna and Kertati, 2024). In environments where collaboration and mutual understanding are essential, such a relationship breakdown is likely to reduce employees’ sense of security concerning interpersonal risk-taking. From the discussion above, the following hypothesis was developed:

*H2:* Silo thinking is significantly and negatively associated with psychological safety.

#### 2.2.3 Relationship between psychological safety and staff engagement

Psychological safety is recognised as a vital relational factor that affects staff engagement, especially in challenging, interrelated settings such as healthcare (O'Donovan and McAuliffe, 2020). Psychological safety is characterised by a collective conviction among employees that they can express their opinions, ask questions, or admit mistakes without apprehension of adverse repercussions, thereby fostering open communication and interpersonal risk-taking (Edmondson, 1999). Social Capital Theory asserts that psychological safety reflects the degree of relational capital within teams, based on trust, reciprocity, and shared standards that promote collaborative behaviour (Edmondson and Lei, 2014; Obeng et al., 2025a). From a Social Exchange Theory perspective, psychologically safe environments foster positive social exchanges that encourage employees to reciprocate supportive workplace relationships through greater engagement (Dhir and Vallabh, 2025).

Although engagement is frequently viewed as a personal motivational condition, research increasingly acknowledges its social context and the influence of relational dynamics (Anyaegbunam et al., 2021). When employees perceive substantial relational capital, encompassing trust and supportive connections, they are more likely to invest cognitive, emotional, and behavioural energy in their work. Psychological safety provides the interpersonal certainty required for commitment, reduces defensive behaviour, and fosters heightened engagement in job activities (Dhir and Vallabh, 2025).

Empirical studies often support this relationship. Meta-analytic evidence indicates that psychological safety is a crucial precursor to professional engagement, as secure conditions enable employees to express themselves authentically and contribute meaningfully (Frazier et al., 2017). Wijerathna and Bandara (2023) similarly revealed that psychological safety positively influences engagement when integrated with various psychological states, highlighting its significance in inspiring employee participation (Obeng et al., 2024). In the context of organisational transition, psychological safety has been demonstrated to foster proactive engagement by allowing employees to express concerns and provide valuable input (Albrecht et al., 2023).

Psychological safety is particularly crucial in interconnected and interrelated healthcare environments (LaPlante et al., 2025). When employees perceive a safe environment for communication and support from colleagues across sectors, relationship coordination improves, increasing commitment and engagement in their work (Silva and Duarte, 2025). Consistent with Social Exchange Theory, employees who perceive trust and support are more likely to reciprocate these positive relational experiences by investing greater effort and commitment in their work. In environments perceived as highly perilous, employees may reduce effort and limit involvement as a self-preservation tactic.

Together, Social Capital Theory explains how psychological safety emerges from strong relational capital, whereas Social Exchange Theory explains why psychologically safe environments promote higher staff engagement. Social Capital Theory suggests that psychological safety enhances staff engagement by reinforcing relational capital (Carmeli, 2007). This enables individuals to focus on their responsibilities without worrying about how their actions affect their interpersonal relationships. From the discussion above, the following hypothesis was developed:

*H3:* Psychological safety is significantly and positively associated with staff engagement.

#### 2.2.4 Relationship between perceived supervisor support and staff engagement

Perceived supervisor support is a significant relational asset in an organisation. Social Capital Theory suggests that supervisors build relational capital by fostering trust, facilitating reciprocity, and promoting shared norms among team members (Xu et al., 2022). Social Exchange Theory further suggests that supportive supervisors establish high-quality exchange relationships in which employees feel obliged to reciprocate favourable treatment through positive work attitudes and behaviours, including greater engagement (Blau, 1964; Eisenberger et al., 2002). As pivotal figures in daily interactions, supervisors shape employees’ perceptions of inclusion, gratitude, and belonging, all of which are essential to maintaining engagement (Karim et al., 2025).

Supportive supervisors demonstrate respect, fairness, and consistency, all of which enhance employees’ confidence in their work environment. Shanock and Eisenberger (2006) suggest that supervisors often serve as organisational representatives, and employees perceive their supportive behaviours as expressions of the organisation’s overarching concern. When supervisors demonstrate trust and support, they build relational capital, increasing the likelihood that employees will invest more mental, emotional, and behavioural energy in their work (Nielsen and Yarker, 2023). According to Kahn's (1990) engagement model, supportive leadership enhances psychological availability and reduces interpersonal risk-taking, enabling individuals to express themselves authentically at work (Colladon and Grippa, 2025).

Empirical studies consistently demonstrate a strong correlation between supervisory support and engagement outcomes. Research indicates that individuals who perceive their managers as supportive exhibit greater commitment, job satisfaction, and engagement (Cheng et al., 2013; Dysvik and Kuvaas, 2013). Studies indicate that supervisory support enhances engagement not only through formal authority but also through relational dynamics that foster trust and reciprocal connections (Zhang and Gao, 2025).

In challenging healthcare settings marked by professional interdependence and structural fragmentation, supervisory relationships may be particularly significant. Managers can maintain staff engagement during challenging periods by fostering transparent, constructive communication (Jiang and Shen, 2023). This fosters a sense of value and connection among employees. Accordingly, Social Capital Theory explains how supervisors strengthen relational capital, whereas Social Exchange Theory explains why employees reciprocate supervisory support with stronger engagement. Social Capital Theory asserts that perceived support from supervisors bolsters staff engagement by reinforcing relational capital and validating individuals’ commitment to their professional responsibilities (Nauman et al., 2024). From the discussion above, the following hypothesis was developed:

*H4:* Perceived supervisor support is significantly and positively associated with staff engagement.

#### 2.2.5 Mediating role of psychological safety

Silo thinking constitutes a structural–relational impediment that hinders communication and collaboration among individuals within healthcare organisations (Kreindler et al., 2012). Social Capital Theory suggests that silo thinking diminishes structural and relational capital by fragmenting networks and undermining trust within professional organisations (Bouwer et al., 2021). This disintegration of connections diminishes employees’ sense of belonging to a collective and undermines their pursuit of a shared objective, adversely affecting the conditions essential for sustained engagement (Imboden, 2024).

Psychological safety is a significant relational mechanism through which these structural conditions may influence staff engagement. Psychological safety, characterised as a collective conviction that the workplace fosters interpersonal risk-taking (Joseph and Seshadri, 2025), includes trust, reciprocity, and constructive interaction, which are fundamental components of relational capital. Silo thinking impedes cross-boundary collaboration and hinders information sharing, making employees less willing to express ideas, seek assistance, or fully participate in their work (Jeske and Olson, 2025). From a Social Exchange Theory perspective, weakened interpersonal relationships reduce employees’ confidence that positive workplace interactions will be reciprocated, thereby lowering psychological safety and subsequent engagement (Xu et al., 2025).

Empirical evidence supports this sequential relationship. Research indicates that siloed workplaces foster isolation and reduce collaboration, adversely affecting employees’ emotional and cognitive engagement (Kreindler et al., 2012). Similarly, psychological safety has consistently been associated with higher levels of work engagement across different organisational settings (Frazier et al., 2017; Wowor and Dewi, 2022). These findings suggest that silo thinking likely affects staff engagement indirectly through employees’ perceptions of psychological safety.

Drawing on both Social Capital Theory and Social Exchange Theory, this study argues that silo thinking weakens the relational conditions necessary for psychological safety. In contrast, reduced psychological safety diminishes employees’ willingness to invest fully in their work. Consequently, psychological safety provides a plausible explanatory mechanism through which silo thinking influences staff engagement (Campos et al., 2025). From the discussion above, the following hypothesis was developed:

*H5:* Psychological safety mediates the relationship between silo thinking and staff engagement.

#### 2.2.6 Moderating role of perceived supervisor support

This study examines the relationships among silo thinking, psychological safety, and staff engagement in healthcare organisations by considering perceived supervisor support as a relational boundary condition. Social Capital Theory suggests that supervisors play a vital role in strengthening structural and relational capital through trust, reciprocity, shared understanding, and supportive interactions (Ray et al., 2023). Through inclusive communication, advocacy, and consistent support, supervisors foster collaborative relationships that help employees remain connected despite organisational fragmentation (Duan et al., 2023). Complementing this perspective, Social Exchange Theory proposes that supportive supervisors cultivate high-quality exchange relationships that encourage employees to reciprocate organisational support through positive workplace attitudes and behaviours (Blau, 1964).

Silo thinking diminishes staff engagement by weakening collaboration, shared goals, and relational cohesion (Jones et al., 2024; Jin and McDonald, 2017). Supportive supervisors can buffer these adverse effects by reinforcing trust, recognising employees’ contributions, and maintaining positive interpersonal relationships. Consequently, employees who perceive high levels of supervisor support may remain engaged even when organisational structures become fragmented. Conversely, when supervisory support is limited, silo thinking is likely to have a more pronounced negative effect on engagement because employees receive little relational reinforcement (Kyambade et al., 2024; de Waal and van den Berg, 2025).

Silo thinking further disrupts communication and trust, thereby reducing psychological safety (Remtulla et al., 2021). Supervisor support may weaken this adverse relationship by encouraging employees to express concerns, validating their opinions, and demonstrating inclusive leadership behaviours (Yasin et al., 2023). Consistent with Social Exchange Theory, employees with supportive supervisory relationships are more likely to view interpersonal interactions as trustworthy and mutually beneficial, thereby maintaining psychological safety despite structural fragmentation.

Psychological safety enhances staff engagement by enabling employees to participate confidently without fear of negative interpersonal consequences (Edmondson and Bransby, 2023). This positive relationship is expected to strengthen when employees perceive greater supervisor support, as supervisors reinforce employees’ confidence, recognition, and sense of belonging. Conversely, psychologically safe environments may not sustain engagement without supervisory support (Smeets et al., 2021).

Drawing on Social Capital Theory and Social Exchange Theory, perceived supervisor support is expected to function as a relational stabiliser that weakens the adverse effects of silo thinking while strengthening the positive influence of psychological safety on staff engagement (Le et al., 2023). From the discussion above, the following hypotheses were developed:

*H6:* Perceived supervisor support moderates the relationship between silo thinking and staff engagement.

*H7:* Perceived supervisor support moderates the relationship between silo thinking and psychological safety.

*H8:* Perceived supervisor support moderates the relationship between psychological safety and staff engagement.

Table 1 provides a summary definition of the study constructs.

**Table 1:** Definition of study’s constructs.

Source	Construct	Definition	Role in model
Jeske and Olson (2025)	Silo thinking	Silo thinking refers to departmental fragmentation characterised by communication barriers, limited information sharing, and weak collaboration across organisational units, often arising when departments operate according to distinct goals and priorities	Independent variable
Kahn (1990), Bakker and Albrecht (2018); Albrecht et al. (2021)	Staff engagement	A positive and fulfilling work-related state characterised by vigour, dedication, and absorption	Dependent variable
Edmondson (1999); Edmondson and Lei (2014)	Psychological safety	Psychological safety is defined as the collective belief that individuals can express their thoughts, ask questions, or acknowledge mistakes without fearing adverse interpersonal consequences	Mediating variable
Eisenberger et al. (2002); Karim et al. (2025)	Perceived supervisor support	Perceived supervisor support refers to employees’ perceptions that their supervisors appreciate their contributions, prioritise their welfare, and offer both emotional and practical assistance	Moderating variable

## 3 Methodology

### 3.1 Research design and approach

This study follows the positivist research paradigm, which asserts that social reality is objective, observable, and measurable, and that quantitative approaches can examine interactions among multiple aspects (Obeng, 2023; Park et al., 2020). This study utilised a quantitative research methodology to examine the relationships among silo thinking, psychological safety, perceived supervisor support, and staff engagement (see Figure 1) within the Ghanaian healthcare system. These concepts are objectively measurable and are analysed using statistical methods appropriate for assessing mediation and moderation effects. This method facilitates systematic data collection, objective measurement, and repeatable analysis. These features enhance the rigour and clarity of the research process. A positivist, quantitative methodology is especially appropriate, given the study’s aim to test theoretically established hypotheses and determine the strength and direction of correlations among variables, rather than to provide inductive or interpretive insights. Yilmaz (2013) asserts that empirical research attains coherence, validity, and credibility when the research paradigm is explicitly linked to methodological choices. The study integrates a positivist framework with a quantitative analytical approach to ensure coherence among its philosophical assumptions, research objectives, and analytical procedures. The positivist approach provides a robust, theoretically coherent framework for examining how structural factors (silo thinking) and relational factors (psychological safety and supervisor support) relate to staff engagement in healthcare organisations. This congruence improves the study’s explanatory power and strengthens the validity of its conclusions within the Ghanaian healthcare setting.

**Figure 1:**
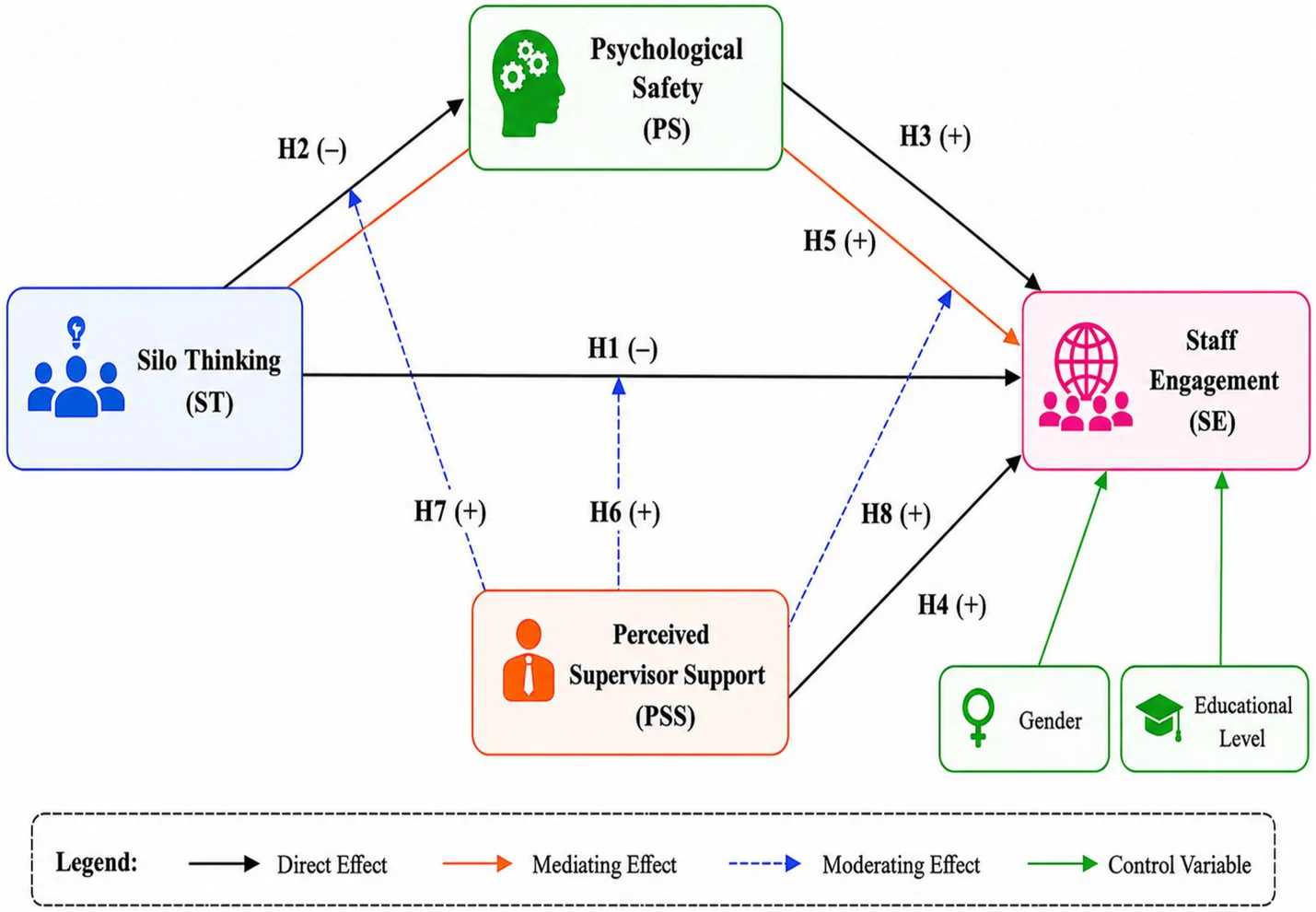
Research model.

### 3.2 Population and sample size

The study focused on public and private hospitals in Ghana’s southern regions, including the Volta, Ashanti, Western, Central, Greater Accra, and Eastern regions. These areas exhibit diverse healthcare landscapes, comprising public and private facilities that serve urban, peri-urban, and rural populations. This range provides a comprehensive understanding of workforce dynamics and healthcare delivery, enabling a broader application of the study’s findings in Ghana.

Because the number of healthcare staff across the selected institutions was unknown, the sample size was initially determined using the Cochran (1977) formula. Cochran’s formula is widely used in survey research to determine the minimum sample size when the target population is unknown or cannot be precisely identified. Consequently, this study adopted it to establish a conservative baseline sample size for data collection. The formula is given as follows:

$$n=\frac{Z^{2}*P*(1-P)}{e^{2}\;}$$

*n* is the required sample size,

Z is the Z-score corresponding to the desired confidence level (1.96),

*P* is the estimated proportion or prevalence of the characteristic being studied in the population (unknown = 0.5).

*e* is the desired margin of error.

$$n=\frac{1.96^{2}}{4{\left(0.05\right)}^{2}\;}$$

$$n=\frac{3.8416}{0.01\;}$$

n=384.16

n=384.16=385

⌈ ⌉ mean “round up to the next integer.

Because the calculated sample size represents the minimum number of participants required, the resulting value was rounded up to the next whole participant using the ceiling function (Goulet and Cousineau, 2019), such that ⌈384.16⌉ = 385. Thus, Cochran’s calculation provided an approximate survey sample-size reference of 384–385 participants rather than a strict data-collection target. Because purposive sampling was employed, this calculation does not imply that the resulting sample is statistically representative of the target population.

Cochran’s formula is primarily designed to determine survey sample size rather than statistical power for structural equation modelling. Therefore, an *a priori* power analysis was performed using G*Power 3.1 to ensure sufficient statistical power for hypothesis testing (Faul et al., 2009; Kang, 2021). The analysis used the most complex structural equation in the hypothesised model, with seven predictors of staff engagement: silo thinking, psychological safety, perceived supervisor support, the two interaction terms (perceived supervisor support × silo thinking and perceived supervisor support × psychological safety), educational level, and sex. A medium effect size (*f*^2^ = 0.15), a significance level (α) of 0.05, and a desired statistical power of 0.95 were specified. The analysis indicated a minimum required sample size of 153 participants (see Appendix A). Data collection continued until 384 completed questionnaires were obtained, consistent with the sample-size estimate established before data collection. Following the initial data screening in Statistical Package for the Social Sciences (SPSS), the listwise deletion procedure was applied to identify and remove questionnaires with incomplete or missing responses. Consequently, 21 questionnaires were excluded, resulting in a final analytical sample of 363 valid responses. This represents a questionnaire retention rate of 94.5%. The final analytical sample substantially exceeded the minimum sample size of 153 participants established through the G*Power analysis and was therefore sufficient to estimate the proposed structural model.

### 3.3 Sampling and data collection technique

Data collection occurred over 9 weeks from February 3 to April 7, 2026. A purposive, multi-stage sampling approach was used. In the initial stage, public and private hospitals in Ghana were purposively chosen from the Volta, Ashanti, Western, Central, Greater Accra, and Eastern regions. A total of 25 hospitals participated to ensure a broad range of institutional types. Hospitals were selected based on their accessibility, willingness to participate, and their ability to provide eligible healthcare professionals from diverse clinical and administrative departments. The participating institutions included teaching hospitals, regional hospitals, district hospitals, and polyclinics. This approach clarified that the goal was to capture diversity in hospital settings, not to reflect their nationwide proportional prevalence. The hospitals were selected solely to obtain a diverse array of individual healthcare practitioners; the hospitals themselves were not treated as units of analysis.

In the second stage, hospital administrators and human resource managers at the selected institutions served as institutional gatekeepers, facilitating access to staff across departments. These gatekeepers facilitated access to eligible participants and coordinated the internal distribution of the survey. At this stage, the sampling approach was purposive, aiming to include as many different departments as possible to capture diversity across institutional units rather than to obtain a statistically representative sample of healthcare institutions or employees. In the final stage, healthcare practitioners from various departments received the surveys. Human resource managers and administrators facilitated access to eligible participants and supported internal distribution of the questionnaire. However, this procedure may have introduced some degree of institutional influence, as the researchers could not determine how potential participants were selected within each hospital. Hospital administrators and human resource managers distributed the survey link and QR code digitally via the computer-assisted web interviewing (CAWI) technique. They shared the questionnaire internally with eligible healthcare practitioners. Because the questionnaire was distributed internally, the exact number of individuals who received the invitation from each hospital or department could not be definitively determined. Consequently, the actual response rate could not be calculated, and the potential for selection bias could not be fully assessed.

Specific inclusion and exclusion criteria were established to ensure that the selected participants aligned with the study’s objectives. The 25 participating healthcare facilities comprised district hospitals, regional hospitals, teaching hospitals, and polyclinics, as they provide a range of healthcare services and facilitate interactions across various healthcare professions and organisational structures. Polyclinics are larger primary healthcare facilities that provide multiple outpatient services and accommodate several healthcare professional groups. In contrast, the clinics excluded from the study were stand-alone clinics with a more limited range of services and organisational structure. Participants were excluded if they were national service personnel, attachment staff, contract employees, or had less than 7 months of service with the organisation. Stand-alone clinics and Community-based Health Planning and Services (CHPS) compounds were excluded from the study. The research concentrated on healthcare employees, including administrative and support staff, doctors, nurses, pharmacists, radiographers, physiotherapists, biomedical scientists, and nutritionists. Conservancy labourers, orderly staff, artisans, and mortuary attendants were excluded.

Before data collection, the authors obtained ethical approval from their institution’s Ethics Review Board. The Board reviewed and approved the study protocol, survey instrument, participant recruitment procedures, and informed consent process to ensure compliance with established ethical standards for research involving human participants. In addition, the researchers obtained administrative permission from the management of all participating public and private hospitals in Ghana before administering the survey. Participation was voluntary, and researchers obtained electronic informed consent from all respondents before they completed the questionnaire. Participants were informed of the study’s purpose, assured that their responses would remain anonymous and confidential, advised that they could withdraw at any time without penalty, and told that participation involved no foreseeable risks. All research procedures followed the ethical principles governing research involving human participants.

No personal information was collected, ensuring privacy and confidentiality. The study upheld anonymity during data collection and processing. Because of the hierarchical nature of healthcare organisations, we implemented specific strategies to mitigate power imbalances and reduce perceived coercion.

Hospital administrators and human resource managers were cautious in disseminating the survey link. This helped avoid any perception of coercion. Participants were assured that their responses would remain anonymous, would not be disclosed to supervisors or management, and would be used exclusively for academic research. We acknowledge that the constructs examined in our study are relationship-oriented. Psychological safety, silo thinking, and perceived supervisory support are deeply embedded in interpersonal relationships and organisational structure. Healthcare environments exhibit considerable power imbalances and professional hierarchies. In these environments, individuals may experience subtle factors that shape their views and how they report workplace dynamics.

Despite assurances of confidentiality, participants may have remained doubtful when asked to assess their supervisors’ support or the effectiveness of their institutional ties. The research recognises that structural realities and other factors influence perceptions of safety, support, and engagement. These factors include professional identity, organisational standards, and contextual power dynamics. Understanding these relationships is ethically necessary when conducting relational research in challenging healthcare settings.

Table 2 presents participants’ demographic characteristics. Out of 363 participants, 208 (57.3%) were male, and 155 (42.7%) were female. In terms of age distribution, 159 participants (43.8%) were aged 31–40 years, 115 (31.7%) were aged 20–30 years, 67 (18.5%) were aged 41–50 years, and 22 (6.1%) were 51 years or older. Regarding educational level, 126 participants (34.7%) obtained a diploma, 103 (28.4%) earned a bachelor’s degree, 71 (19.6%) had a master’s degree, and 63 (17.4%) had a PhD. By position, 201 participants (55.4%) were senior staff, 128 (35.3%) were junior staff, and 34 (9.4%) held top management positions. Concerning organisational tenure, 149 participants (41.0%) had been employed for 1–5 years, 97 (26.7%) for 6–10 years, 78 (21.5%) for 11–15 years, 29 (8.0%) for 16–20 years, and 10 (2.8%) for 21 years or more.

**Table 2:** Demographic characteristics of respondents.

Variable	Category	Frequency	Percentage (%)
Sex	Male	208	57.3
	Female	155	42.7
Age	20–30 years	115	31.7
	31–40 years	159	43.8
	41–50 years	67	18.5
	51 years and above	22	6.1
Educational level	Diploma	126	34.7
	Bachelor’s degree	103	28.4
	Master’s degree	71	19.6
	PhD	63	17.4
Position	Top management	34	9.4
	Senior staff	201	55.4
	Junior staff	128	35.3
Organisational tenure	1–5 years	149	41.0
	6–10 years	97	26.7
	11–15 years	78	21.5
	16–20 years	29	8.0
	21 years and above	10	2.8

### 3.4 Measurement and scale

The measurement instruments used in this study were adopted from previously validated scales to assess the constructs investigated (see Appendix B). The scales were applied in the Ghanaian healthcare context, with contextual wording adjustments made where necessary while retaining the conceptual meaning of the original items. For the psychological safety scale, we adapted references to “team” to “organisation” to align the items with the study’s organisational focus. We made minor wording adjustments to ensure contextual clarity. All questionnaire items were administered in English, the official language and main mode of professional communication in Ghana’s healthcare sector; therefore, no translation or back-translation procedures were required. The instruments have been extensively validated in organisational behaviour and management research. Accordingly, we did not conduct independent pilot testing or expert evaluation before the main survey. All items were evaluated using a 5-point Likert scale from 1 (strongly disagree) to 5 (strongly agree) (Arhinful et al., 2026; Birinci et al., 2025). The single negatively worded question in the perceived supervisor support scale was reverse-coded during data preparation before statistical analysis to ensure that higher scores consistently indicated higher levels of the construct. Each instrument was chosen for its relevance and established psychometric properties in similar study settings.

#### 3.4.1 Silo thinking scale

Silo thinking was measured using 31 items adapted from de Waal et al. (2019) and de Waal and van den Berg (2025), covering values, collaborative operating model, collaborative environment, leadership, and people reward and development, with Cronbach’s alpha coefficients ranging from 0.853 to 0.939 (de Waal et al., 2019). Because the original items were positively framed around practices that reduce organisational silos, the items were reverse-coded before analysis so that higher scores consistently represented greater silo thinking. This recoding ensured that the construct direction aligned with the study’s hypotheses and interpretation. In Ghana’s healthcare environments, silo thinking can hinder knowledge sharing and cross-functional collaboration, both of which are important for effective healthcare delivery.

Conceptually, silo thinking was defined as an overarching organisational orientation reflected across multiple organisational domains, rather than as a construct formed by aggregating independent components. The five dimensions—values, collaborative operating model, collaborative environment, leadership, and people reward and development—represent domain-specific expressions of the same underlying silo orientation. Thus, an organisation characterised by stronger silo thinking would be expected to exhibit a consistent pattern across these domains, including weaker collaborative values, less integrated operating arrangements, a less collaborative organisational environment, leadership behaviours that provide limited support for cross-unit collaboration, and reward and development practices that are less conducive to collaborative working.

Although the five dimensions capture different organisational domains and therefore differ in content, they are theoretically expected to covary because they arise from the same underlying organisational orientation. In other words, changes in silo thinking are expected to be reflected simultaneously across dimensions rather than occurring independently in only one domain. Consequently, the dimensions are treated as reflective manifestations of the higher-order construct rather than formative ingredients that combine to define silo thinking. Although each dimension contributes to the breadth of the construct’s empirical representation, omitting one dimension would reduce content coverage rather than alter the conceptual meaning of silo thinking itself. On this theoretical basis, silo thinking was modelled as a reflective–reflective hierarchical construct.

#### 3.4.2 Psychological safety scale

Psychological safety was measured using the 5-item scale developed by Edmondson (1999), which reported a Cronbach’s alpha of 0.710. In Ghanaian healthcare workplaces, hierarchical norms may discourage open communication, making psychological safety particularly important for encouraging employees to express ideas, voice concerns, and seek assistance without fear of negative consequences.

#### 3.4.3 Staff engagement scale

Staff engagement was measured using the 9-item Utrecht Work Engagement Scale (UWES-9) developed by Schaufeli and Bakker (2004). The scale assesses three dimensions of engagement: vigour, dedication, and absorption, and has demonstrated good internal consistency (Cronbach’s alpha = 0.880). Work engagement is particularly important in Ghana’s healthcare sector because engaged employees are more committed, resilient, and motivated to deliver high-quality patient care.

#### 3.4.4 Perceived supervisor support scale

Perceived supervisor support was measured using five items drawn indirectly from the perceived supervisor support measure reported by Siddiqi et al. (2025), which was based on measures from Rhoades et al. (2001) and Wong (2018). The items assessed supervisors’ concern for employees’ opinions and wellbeing, consideration of employees’ goals and values, willingness to assist, and general concern for employees. The negatively worded item, “My supervisor shows very little concern for me,” was reverse-coded before statistical analysis. The five-item measure demonstrated satisfactory reliability and convergent validity, as reported in Table 3.

**Table 3:** Reliability and validity results (LOC).

Construct/items	Standardised loadings	VIF	α	CR	AVE
Silo thinking					
COE1	0.856	2.758	0.914	0.930	0.625
COE2	0.830	2.425			
COE3	0.784	2.049			
COE4	0.785	2.075			
COE5	0.798	2.149			
COE6	0.761	1.915			
COE7	0.795	2.111			
COE8	0.708	1.676			
COM1	0.835	2.655	0.920	0.933	0.560
COM2	0.844	2.771			
COM3	0.794	2.260			
COM4	0.777	2.102			
COM5	0.763	2.008			
COM6	0.769	2.074			
COM7	0.742	1.933			
COM8	0.706	1.744			
COM9	0.701	1.764			
COM10	0.643	1.554			
COM11	0.623	1.491			
LED1	0.908	2.389	0.842	0.905	0.760
LED2	0.872	2.773			
LED3	0.835	1.800			
PRD1	0.864	2.498	0.880	0.913	0.678
PRD2	0.848	2.262			
PRD3	0.849	2.339			
PRD4	0.800	1.921			
PRD5	0.750	1.663			
VAL1	0.862	2.101	0.841	0.893	0.677
VAL2	0.851	2.020			
VAL3	0.818	1.858			
VAL4	0.757	1.581			
Staff engagement					
ABS1	0.899	2.177	0.817	0.891	0.732
ABS2	0.855	1.891			
ABS3	0.811	1.633			
DED1	0.882	1.970	0.814	0.890	0.729
DED2	0.847	1.763			
DED3	0.832	1.719			
VIR1	0.906	2.357	0.83	0.898	0.747
VIR2	0.841	1.788			
VIR3	0.844	1.884			
Psychological safety					
PS1	0.886	2.851	0.882	0.914	0.681
PS2	0.846	2.334			
PS3	0.827	2.124			
PS4	0.799	1.906			
PS5	0.762	1.738			
Perceived supervisor support					
PSS1	0.888	2.875	0.881	0.913	0.679
PSS2	0.867	2.531			
PSS3	0.817	2.010			
PSS4	0.777	1.815			
PSS5	0.763	1.769			

α, Cronbach’s alpha; CR, composite reliability; AVE, average variance extracted; VIF, variance inflation factor; COE, collaborative environment; COM, collaborative operating model; LED, leadership; PRD, people reward and development; VAL, values; ABS, absorption; DED, dedication; VIR, vigour; PS, psychological safety; PSS, perceived supervisor support.

#### 3.4.5 Control variable

Sex and educational level were incorporated as control variables in the evaluation of staff engagement. Both variables were incorporated into the structural model as direct pathways to the dependent variable to address potential demographic effects. Previous studies show these factors influence engagement-related beliefs, verbal conduct, and psychological safety in organisations (Kahn, 1990). Educational level also indicates differences in professional socialisation and expectations for involvement and supervisory support (Schaufeli et al., 2006). Work experience was excluded to keep the model simple and to avoid overlap with psychological safety and engagement, which could make their individual contributions indistinct. Methodological research warns against using control variables without a strong theory, as this can lead to multicollinearity and mask effects (Becker, 2005; Bernerth and Aguinis, 2016). This approach follows optimal practices for partial least squares structural equation modelling (PLS-SEM) (Hair and Alamer, 2022).

### 3.5 Statistical tools

The data in this research were analysed using SPSS version 24 and PLS-SEM version 4.1.1.4. PLS-SEM is suitable for evaluating complex models with many components, particularly when the sample size is moderate, and the data do not fully meet traditional SEM assumptions (Sarstedt et al., 2022). This technique is well suited to analysing relationships among constructs because it facilitates the concurrent estimation of the measurement and structural models. The tool supports a thorough evaluation of the proposed theoretical framework by providing detailed information on path coefficients, model fit, and predictive significance. The measurement model was assessed in PLS-SEM using SmartPLS 4. The assessment examined indicator loadings, internal consistency reliability (Cronbach’s alpha and composite reliability [CR]), convergent validity (average variance extracted [AVE]), and discriminant validity (heterotrait–monotrait [HTMT] ratio). We tested hypotheses using bootstrapping with 5,000 resamples and the bias-corrected and accelerated (BCa) bootstrap method. Given the directional nature of the hypotheses, one-tailed tests were conducted at a 5% significance level (α = 0.05). Accordingly, the 5th and 95th percentile BCa bootstrap limits form a two-sided 90% interval, which corresponds to a one-tailed test at α = 0.05. We reported these bootstrap limits to assess the direction and statistical significance of the hypothesised relationships. For positive hypotheses, we considered results significant when the lower bootstrap limit was above zero; for negative hypotheses, we considered results significant when the upper bootstrap limit was below zero.

## 4 Results and findings

### 4.1 Descriptive statistics and matrix correlation

Table 4 presents the descriptive statistics and correlation matrix for the variables under investigation. The findings indicate that the evaluated healthcare facilities exhibit moderate levels of silo thinking (M = 3.07, SD = 0.59), staff engagement (M = 2.83, SD = 0.55), psychological safety (M = 2.79, SD = 0.46), and perceived supervisory support (M = 2.88, SD = 0.69). The average ratings indicate that, despite silo structures within organisations, staff experience only a moderate sense of engagement and security. This pattern suggests the environment is neither severely disorganised nor ideally integrated, suggesting that structural modelling can take various forms.

**Table 4:** Descriptive statistics and matrix correlation.

Descriptive statistics matrix correlation							
Construct	*N*	Mean	SD	1	2	3	4
Silo thinking (1)	363	3.073	0.587	1			
Staff engagement (2)	363	2.833	0.545	−0.439**	1		
Psychological safety(3)	363	2.789	0.457	−0.499**	0.699**	1	
Perceived supervisor support (4)	363	2.881	0.687	−0.022	0.500**	0.483**	1

*N* = 363. SD, standard deviation. Correlation coefficients are Pearson’s *r*. **p < 0.01 (two-tailed).

The correlation statistics offer an initial insight into the relationships among the constructs. Silo thinking exhibits a significant negative correlation with staff engagement (*r* = −0.439, *p* < 0.01) and psychological safety (*r* = −0.499, *p* < 0.01). This indicates that high departmental fragmentation is associated with a diminished sense of safety when expressing concerns and a reduced psychological commitment to work. Prolonged communication issues and departmental isolation can lead employees to become disengaged, both cognitively and emotionally, from their work. A significant positive correlation exists between psychological safety and staff engagement (*r* = 0.699, *p* < 0.01). Individuals who feel secure in addressing issues and participating in decision-making are more inclined to exhibit energy, determination, and concentration in their work. This significant correlation is the initial indication that the proposed mediating mechanism may be plausible. Perceived supervisor support shows a significant, positive correlation with both staff engagement (*r* = 0.500, *p* < 0.01) and psychological safety (*r* = 0.483, *p* < 0.01). This indicates that supportive supervisors may enhance employees’ confidence and engagement. Significantly, perceived supervisor support exhibits no substantial correlation with silo thinking (*r* = −0.022), suggesting that structural fragmentation and supervisory behaviour may represent distinct aspects of the organisational environment rather than as interconnected phenomena.

All correlations fall below the threshold typically associated with multicollinearity, indicating that although the constructs are significantly related, they remain empirically distinct and suitable for subsequent analyses.

### 4.2 Measurement model

The measurement model was assessed using a two-stage hierarchical component modelling (HCM) approach (Zola and Perkasa, 2026) in SmartPLS 4. Consistent with the conceptualisation presented in the measurement section, silo thinking was specified as a multidimensional higher-order construct comprising five first-order dimensions (values, collaborative operating model, collaborative environment, leadership, and people reward and development). In contrast, staff engagement was modelled as a higher-order construct reflected by absorption, dedication, and vigour. Both higher-order constructs were specified as reflective–reflective hierarchical component models. Following the recommendations of Hair and Alamer (2022), latent variable scores from the first-order constructs were estimated in the first stage and subsequently used as indicators of the higher-order constructs in the second stage.

#### 4.2.1 Reliability and validity analysis

Table 3 and Figure 2 present the reliability and validity findings for the first-order constructs. The standardised outer loadings indicate that almost all indicators exceeded the recommended threshold of 0.70 (Hair et al., 2021). Two indicators in the collaborative operating model dimension (COM10 = 0.643 and COM11 = 0.623) were slightly below 0.70; however, they exceeded the acceptable threshold of 0.60 suggested for established scales based on theoretical justification (Chin, 1998; Hair et al., 2021). These indicators were consequently retained, as their removal did not significantly enhance composite reliability or AVE, while they contributed to maintaining the conceptual breadth of the collaborative operating model dimension.

**Figure 2:**
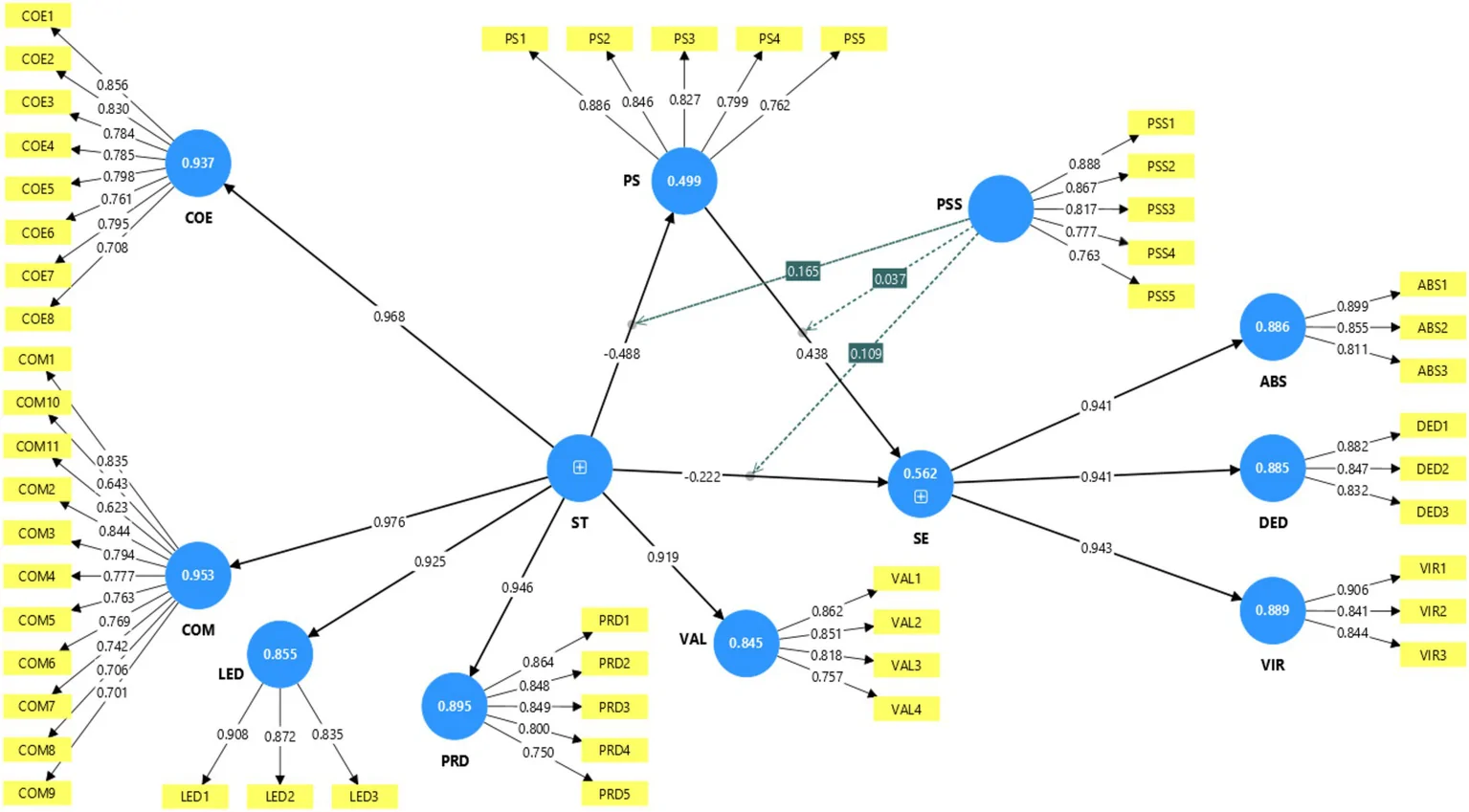
Measurement model.

The variance inflation factor (VIF) values reported in Table 3 ranged from 1.491 to 2.875 across the measurement indicators. All values were below the commonly applied threshold of 3.3, indicating that the measurement indicators did not exhibit problematic levels of collinearity.

Cronbach’s alpha scores ranged from 0.814 to 0.920, exceeding the suggested threshold of 0.70 and therefore indicating satisfactory internal consistency. Similarly, CR scores ranged from 0.890 to 0.933, exceeding the suggested minimum of 0.70 and remaining below the maximum of 0.95, indicating adequate construct reliability without evidence of indicator redundancy (Hair et al., 2021). Convergent validity was evaluated using AVE, with all constructs ranging from 0.560 to 0.760, exceeding the suggested minimum of 0.50 (Fornell and Larcker, 1981). These results collectively support adequate indicator reliability, internal consistency, and convergent validity for all first-order constructs.

The findings on reliability and convergent validity for the higher-order constructs are presented in Table 5 and Figure 3. Silo thinking had a Cronbach’s alpha of 0.972, a composite reliability of 0.978, and an AVE of 0.900. In contrast, staff engagement demonstrated a Cronbach’s alpha of 0.936, a composite reliability of 0.957, and an AVE of 0.887. The higher-order loadings ranged from 0.929 to 0.966 for silo thinking dimensions and from 0.941 to 0.943 for staff engagement dimensions, demonstrating a robust relationship between the higher-order constructs and their corresponding lower-order dimensions. Although the reliability coefficients for the higher-order constructs are relatively higher than those for the first-order constructs, this outcome is theoretically anticipated in a reflective–reflective hierarchical component model assessed by the two-stage method. The higher-order constructs are represented by latent variable scores obtained from reliable first-order constructs rather than individual questionnaire items (Wetzels et al., 2009). As a result, the random measurement error linked to specific indicators is significantly reduced, while the common variance shared among the lower-order dimensions is retained. This aggregation inherently produces higher estimates of internal consistency and convergent validity, without indicating redundancy among the lower-order constructs. Consequently, the high Cronbach’s alpha, CR, and AVE values for the higher-order constructs reflect the strong conceptual consistency of their underlying dimensions and empirically validate the adequacy of the hierarchical measurement model.

**Table 5:** Reliability and validity results (HOC).

Construct/items	Standardised loadings	α	CR	AVE
Silo thinking		0.972	0.978	0.900
COE	0.961			
COM	0.966			
LED	0.938			
PRD	0.948			
VAL	0.929			
Staff engagement		0.936	0.959	0.887
ABS	0.943			
DED	0.941			
VIR	0.941			

α, Cronbach’s alpha; CR, composite reliability; AVE, average variance extracted; COE, collaborative environment; COM, collaborative operating model; LED, leadership; PRD, people reward and development; VAL, values; ABS, absorption; DED, dedication; VIR, vigour.

**Figure 3:**
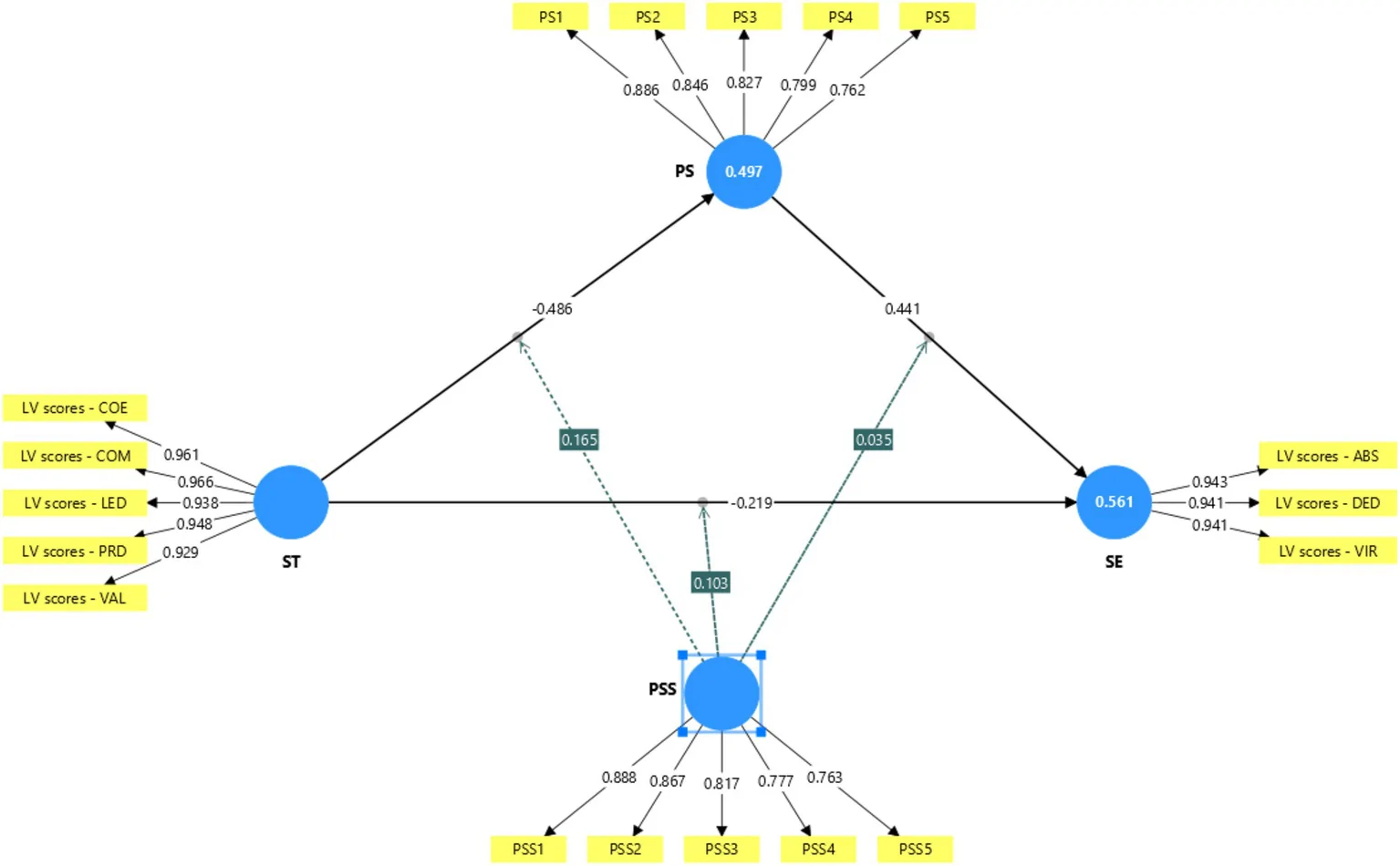
HOC loadings.

#### 4.2.2 Discriminant validity

Discriminant validity for the higher-order constructs was evaluated using the HTMT ratio, which is considered a more stringent standard than the Fornell–Larcker criterion or cross-loadings for assessing discriminant validity in PLS-SEM (Henseler et al., 2015; Hair and Alamer, 2022). The structural model was assessed using higher-order constructs, and discriminant validity was examined at this level to ensure that the latent variables in the structural model were empirically distinct.

According to Table 6, all HTMT values fell below the stringent threshold of 0.85, indicating satisfactory discriminant validity among the higher-order constructs. The highest HTMT value recorded was 0.769 for the relationship between psychological safety and staff engagement, indicating a relatively strong relationship, while remaining well below the recommended threshold. This finding is theoretically anticipated, as psychological safety is a crucial antecedent of staff engagement. The remaining HTMT values were 0.548 for psychological safety and perceived supervisor support, 0.552 for perceived supervisor support and staff engagement, 0.540 for psychological safety and silo thinking, 0.461 for staff engagement and silo thinking, and 0.035 for perceived supervisor support and silo thinking. These findings indicate that each higher-order construct captures a distinct conceptual domain, notwithstanding the theoretical relationship between them. The HTMT analysis supports the conclusion that the higher-order constructs exhibit adequate discriminant validity, thereby reinforcing the validity of the hierarchical measurement model and the subsequent structural-model analysis.

**Table 6:** Discriminant validity results (HTMT ratio).

Construct	PS	PSS	SE	ST
PS				
PSS	0.548			
SE	0.769	0.552		
ST	0.540	0.035	0.461	

PS, psychological safety; PSS, perceived supervisor support; SE, staff engagement; ST, silo thinking.

### 4.3 Structural model

Before assessing the structural model, Harman’s single-factor test and VIF analysis were used to assess common method bias. Furthermore, the model fit was evaluated to assess whether the proposed model adequately represented the observed data and provided a suitable basis for hypothesis testing. The effect size (*f*^2^) was examined to determine how much each external component explains the internal structures. The coefficient of determination (*R*^2^) was used to assess the model’s explanatory power. This showed the extent to which the independent variables could explain variation in the dependent variables. The blindfolding technique was employed to assess predictive relevance (*Q*^2^), indicating whether the model could forecast the observed data beyond mere statistical fitting. Ultimately, all indicators for the latent variable were significant. Item importance is essential for assessing construct reliability and validity before examining structural links (Hair et al., 2019; Henseler et al., 2015).

#### 4.3.1 Common method bias

Statistically, we assessed common method bias using Harman’s single-factor test and the VIF. First, we conducted Harman’s single-factor analysis (see Appendix C) using principal axis factoring. The first unrotated factor accounted for 40.45% of the total variance, which was below the commonly applied 50% criterion (Podsakoff et al., 2003). Subsequently, following Kock's (2015) full-collinearity approach, we introduced an unrelated, randomly generated variable into the PLS-SEM model as an auxiliary dependent variable. The substantive latent constructs, psychological safety, perceived supervisor support, staff engagement, and silo thinking, were specified as predictors of this random variable. In contrast, the substantive relationships in the original structural model were retained. Full-collinearity VIFs were then obtained for the predictors of the auxiliary variable. The resulting VIF values (see Table 7 and Appendix D) were 1.912 for psychological safety, 1.421 for perceived supervisor support, 2.087 for staff engagement, and 1.428 for silo thinking. All values were below the conservative threshold of 3.3, suggesting that problematic common method bias was unlikely to materially influence the results (Kock, 2015; Diamantopoulos and Siguaw, 2006).

**Table 7:** Full-collinearity test.

Latent construct	VIF
Psychological safety	1.912
Perceived supervisor support	1.421
Staff engagement	2.087
Silo thinking	1.428

#### 4.3.2 Model fit

Table 8 presents the model fit indices for the PLS-SEM model. The standardised root mean square residual (SRMR = 0.041) is below the recommended threshold of 0.08 (Hu and Bentler, 1999), indicating a close correspondence between the observed and model-implied correlation matrices. The normed fit index (NFI = 0.940) exceeds the commonly used threshold of 0.90 (Bentler and Bonett, 1980), indicating better relative fit than the null model. These fit indices, considered alongside the measurement- and structural-model results, provide evidence of satisfactory model fit and support the proposed model’s adequacy for examining the relationships among silo thinking, psychological safety, staff engagement, and perceived supervisor support.

**Table 8:** Model fit.

Indices	Estimated model
SRMR	0.041
NFI	0.940

SRMR, standardised root mean square residual; NFI, normed fit index.

#### 4.3.3 Effect size (*f*^2^)

Table 9 displays the effect sizes (*f*^2^) for the structural relationships. Cohen's (2013) criteria classify effect sizes of 0.02, 0.15, and 0.35 as small, medium, and large effects, respectively.

**Table 9:** Effect size (*f*^2^).

Path/Indices	*f* ^2^
Psychological safety → Staff engagement	0.222
Perceived supervisor support → Staff engagement	0.121
Perceived supervisor support → Psychological safety	0.408
Perceived supervisor support × Psychological safety → Staff engagement	0.002
Perceived supervisor support × Silo thinking → Staff engagement	0.017
Perceived supervisor support × Silo thinking → Psychological safety	0.051
Silo thinking → Staff engagement	0.071
Silo thinking → Psychological safety	0.469

The findings indicate that silo thinking has a large effect size on psychological safety (*f*^2^ = 0.469), while perceived supervisor support also has a large effect size on psychological safety (*f*^2^ = 0.408). These findings indicate that both predictors contribute substantially to explaining psychological safety within the structural model.

Psychological safety has a medium effect size for staff engagement (*f*^2^ = 0.222), indicating a meaningful contribution to explaining variation in staff engagement. The direct effects of perceived supervisor support (*f*^2^ = 0.121) and silo thinking (*f*^2^ = 0.071) on staff engagement are small. These findings indicate that both predictors contribute less to explaining staff engagement than psychological safety.

The interaction effects of perceived supervisor support × psychological safety on staff engagement (*f*^2^ = 0.002) and perceived supervisor support × silo thinking on staff engagement (*f*^2^ = 0.017) are negligible. In contrast, the interaction effect of perceived supervisor support × silo thinking on psychological safety (*f*^2^ = 0.051) has a small effect size. Although these interaction effects are relatively small, they provide additional information about the conditional relationships specified in the structural model. Overall, the *f*^2^ results indicate that psychological safety has a medium effect size for staff engagement, whereas silo thinking and perceived supervisor support have large effect sizes for psychological safety and small effect sizes for staff engagement.

#### 4.3.4 Coefficient of determination (*R*^2^) and predictive relevance (*Q*^2^)

Table 10 presents the coefficient of determination (*R*^2^) and predictive relevance (*Q*^2^) values for the endogenous constructs. The *R*^2^ value for staff engagement is 0.561, indicating that silo thinking, psychological safety, perceived supervisor support, and interaction variables account for 56.1% of the variance in staff engagement. *R*^2^ values of 0.75, 0.50, and 0.25 are deemed substantial, moderate, and weak, respectively, based on commonly accepted criteria (Hair et al., 2019). The observed *R*^2^ value of 0.561 falls within the moderate range, indicating that the model explains a moderate proportion of the variance in staff engagement among the sampled healthcare professionals. The *R*^2^ value for psychological safety is 0.497, indicating that silo thinking and perceived supervisor support explain 49.7% of the variance in psychological safety. This value indicates explanatory power close to the moderate benchmark of 0.50. The finding suggests that silo thinking and perceived supervisor support are important factors associated with employees’ perceptions of safety in expressing their opinions and being transparent at work.

**Table 10:** Coefficient of determination (*R^2^*) and predictive relevance (*Q*^2^).

Construct/indices	*R^2^*	*Q* ^2^
Staff engagement	0.561	0.453
Psychological safety	0.497	0.489

Stone–Geisser’s *Q*^2^ values derived from blindfolding were employed to assess predictive relevance (Geisser, 1973). The *Q*^2^ values for both endogenous variables were positive (psychological safety = 0.489; staff engagement = 0.453), indicating predictive relevance of the model for the endogenous constructs (Chin, 1998). The *Q*^2^ value for psychological safety was higher than that for staff engagement, indicating greater predictive relevance for psychological safety within the assessed model. This pattern is consistent with the model structure, in which psychological safety is positioned as an endogenous construct linking silo thinking and perceived supervisor support to staff engagement. The positive *Q*^2^ values indicate that the model has predictive relevance for the endogenous constructs within the sample analysed.

#### 4.3.5 Latent variable indicators

The findings show that the outer loadings of the indicators reported in Table 11 and Appendix E were statistically significant. For the indicators reported in Table 11, the outer loadings ranged from 0.762 to 0.966, and all estimable indicator loadings were statistically significant at *p* < 0.001, with their bootstrap limits remaining above zero. This range applies to the indicators reported in the table and does not represent the full range of loadings across all first-order measurement items. In particular, the first-stage assessment reported in Table 3 included COM10 (0.643) and COM11 (0.623), which were retained based on the measurement criteria discussed previously. According to Hair et al. (2021) and Obeng et al. (2025b), the magnitude of the outer loadings provides evidence of indicator reliability, with the results indicating that the indicators adequately represent their respective latent variables. Convergent validity was assessed primarily through the magnitude of the outer loadings, composite reliability, and AVE, rather than through statistical significance alone (Fornell and Larcker, 1981). Together, these results provide evidence of satisfactory measurement quality and support subsequent assessment of the structural relationships.

**Table 11:** Latent variable indicators.

Indicator	Outer loading (O)	*t*-statistic	*p*-value	5.0%	95.0%
LV scores – ABS ← SE	0.943	166.929	<0.001	0.933	0.952
LV scores – COE ← ST	0.961	222.039	<0.001	0.954	0.968
LV scores – COM ← ST	0.966	279.799	<0.001	0.961	0.972
LV scores – DED ← SE	0.941	143.079	<0.001	0.929	0.951
LV scores – LED ← ST	0.938	151.210	<0.001	0.928	0.948
LV scores – PRD ← ST	0.948	162.324	<0.001	0.937	0.957
LV scores – VAL ← ST	0.929	131.196	<0.001	0.917	0.940
LV scores – VIR ← SE	0.941	159.768	<0.001	0.931	0.951
PS1 ← PS	0.886	78.287	<0.001	0.867	0.904
PS2 ← PS	0.846	55.238	<0.001	0.820	0.870
PS3 ← PS	0.827	43.088	<0.001	0.793	0.856
PS4 ← PS	0.799	38.312	<0.001	0.763	0.831
PS5 ← PS	0.762	30.760	<0.001	0.719	0.800
PSS1 ← PSS	0.888	75.130	<0.001	0.868	0.906
PSS2 ← PSS	0.867	63.321	<0.001	0.844	0.889
PSS3 ← PSS	0.817	40.493	<0.001	0.782	0.848
PSS4 ← PSS	0.777	31.456	<0.001	0.732	0.813
PSS5 ← PSS	0.763	30.823	<0.001	0.720	0.800

### 4.4 Hypothesis testing

Table 12 presents the hypothesis-testing results. Consistent with the one-tailed testing approach described in the Methods section, the reported 5th and 95th percentile BCa bootstrap limits represent a two-sided 90% interval corresponding to α = 0.05 for the directional hypotheses. Figure 4 presents the structural model, Figures 5, 6 illustrate the significant interaction effects, and Figure 7 presents the non-significant interaction effect. Seven of the eight proposed hypotheses were supported.

**Table 12:** Hypothesis testing.

Hypothesis	Path	*β*	*M*	SD	*t*-value	BCa bootstrap limits (5th, 95th Percentiles)	*p*-value	Decision
Direct effects								
H1	ST → SE	−0.219	−0.219	0.046	4.802	[−0.293, −0.145]	<0.001	Supported
H2	ST → PS	−0.486	−0.486	0.034	14.178	[−0.541, −0.430]	<0.001	Supported
H3	PS → SE	0.441	0.441	0.049	8.908	[0.358, 0.521]	<0.001	Supported
H4	PSS → SE	0.275	0.276	0.041	6.697	[0.207, 0.344]	<0.001	Supported
	PSS → PS	0.455	0.456	0.037	12.319	[0.395, 0.516]	<0.001	-
Indirect effect								
H5	ST → PS → SE	−0.214	−0.214	0.030	7.238	[−0.264, −0.166]	<0.001	Supported
Moderating effects								
H6	PSS × ST → SE	0.103	0.103	0.041	2.529	[0.036, 0.170]	0.006	Supported
H7	PSS × ST → PS	0.165	0.162	0.035	4.640	[0.103, 0.221]	<0.001	Supported
H8	PSS × PS → SE	0.034	0.035	0.041	0.844	[−0.031, 0.103]	0.199	Not supported
Control variables								
EL	EL → SE	0.096	0.097	0.035	2.777	[0.041, 0.154]	0.003	
Sex	Sex →SE	0.051	0.051	0.070	0.729	[−0.063, 0.170]	0.233	

ST, silo thinking; PS, psychological safety; PSS, perceived supervisor support; SE, staff engagement; EL, educational level; β, standardised path coefficient; M, bootstrap sample mean; SD, bootstrap standard deviation; BCa, bias-corrected and accelerated. Hypotheses were tested using one-tailed tests at α, 0.05 because of their directional formulation. The 5th and 95th percentile BCa bootstrap limits represent a two-sided 90% interval corresponding to the one-tailed α, 0.05 criterion. The PSS → PS path was included as a non-hypothesised main effect to ensure appropriate estimation of the PSS × ST → PS interaction; therefore, no hypothesis decision is reported for this path. For positive hypotheses, significance was established when the lower limit was above zero; for negative hypotheses, significance was established when the upper limit was below zero.

**Figure 4:**
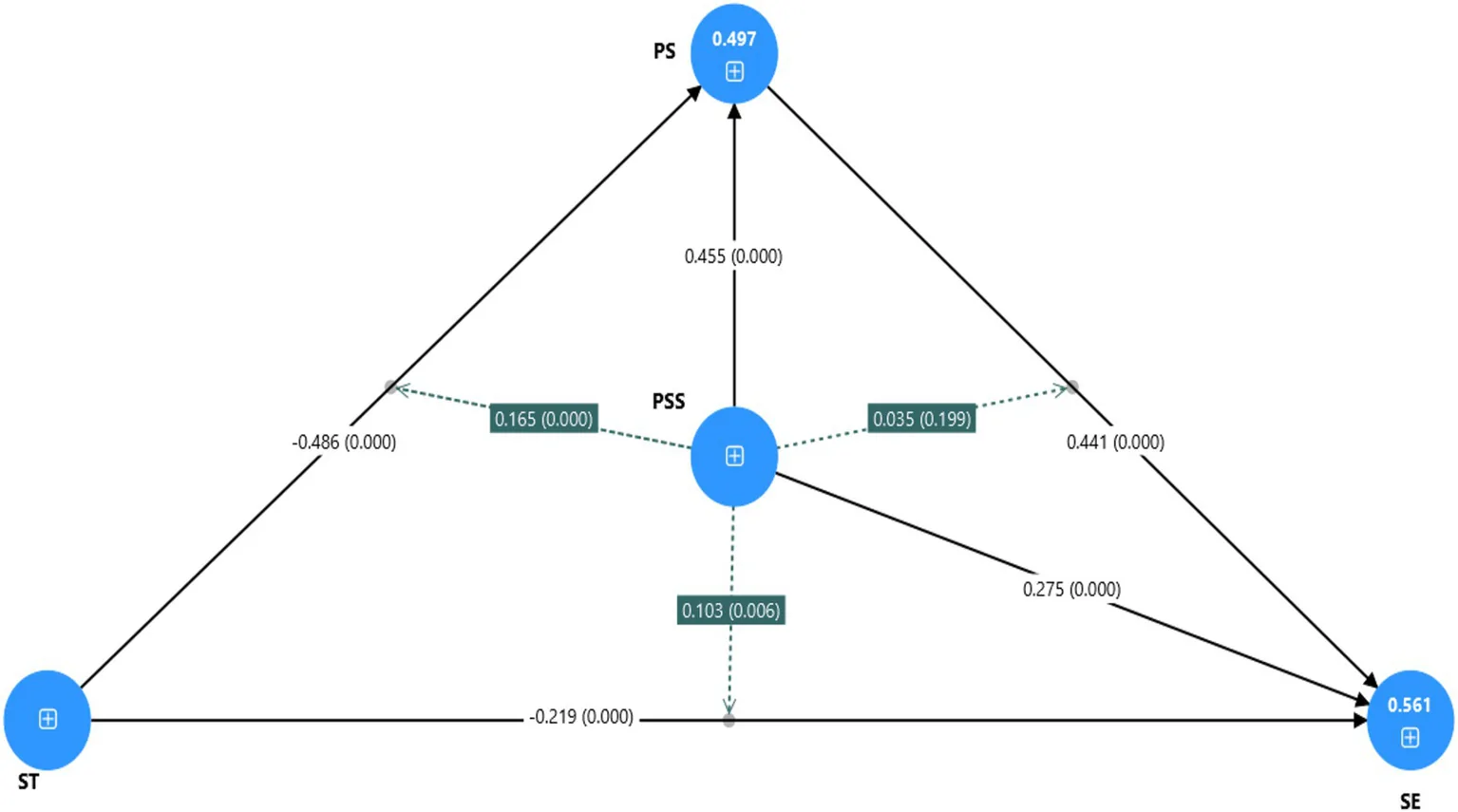
Structural model.

**Figure 5:**
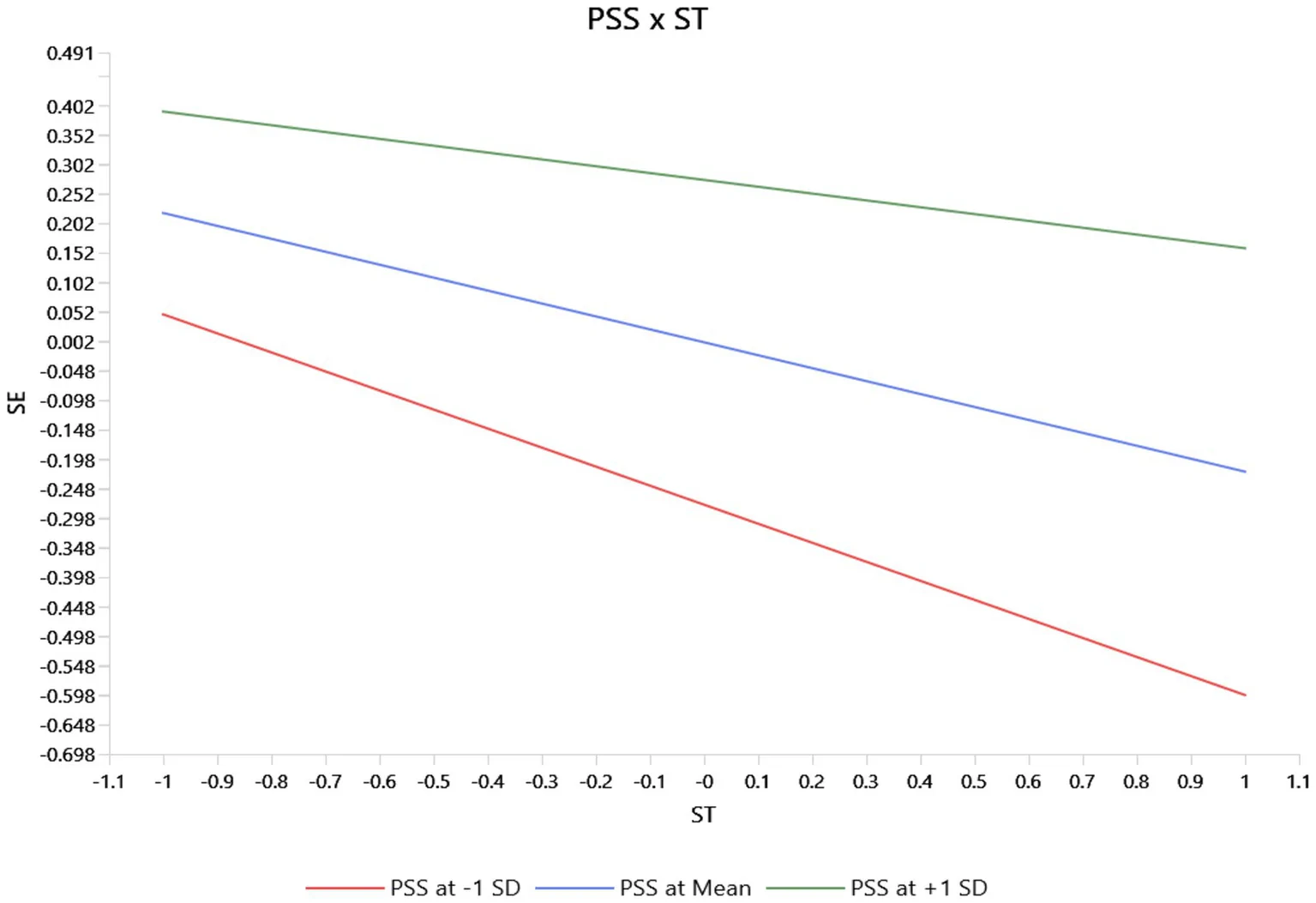
Moderating effect of perceived supervisor support on the relationship between silo thinking and staff engagement (PSS × ST → SE).

**Figure 6:**
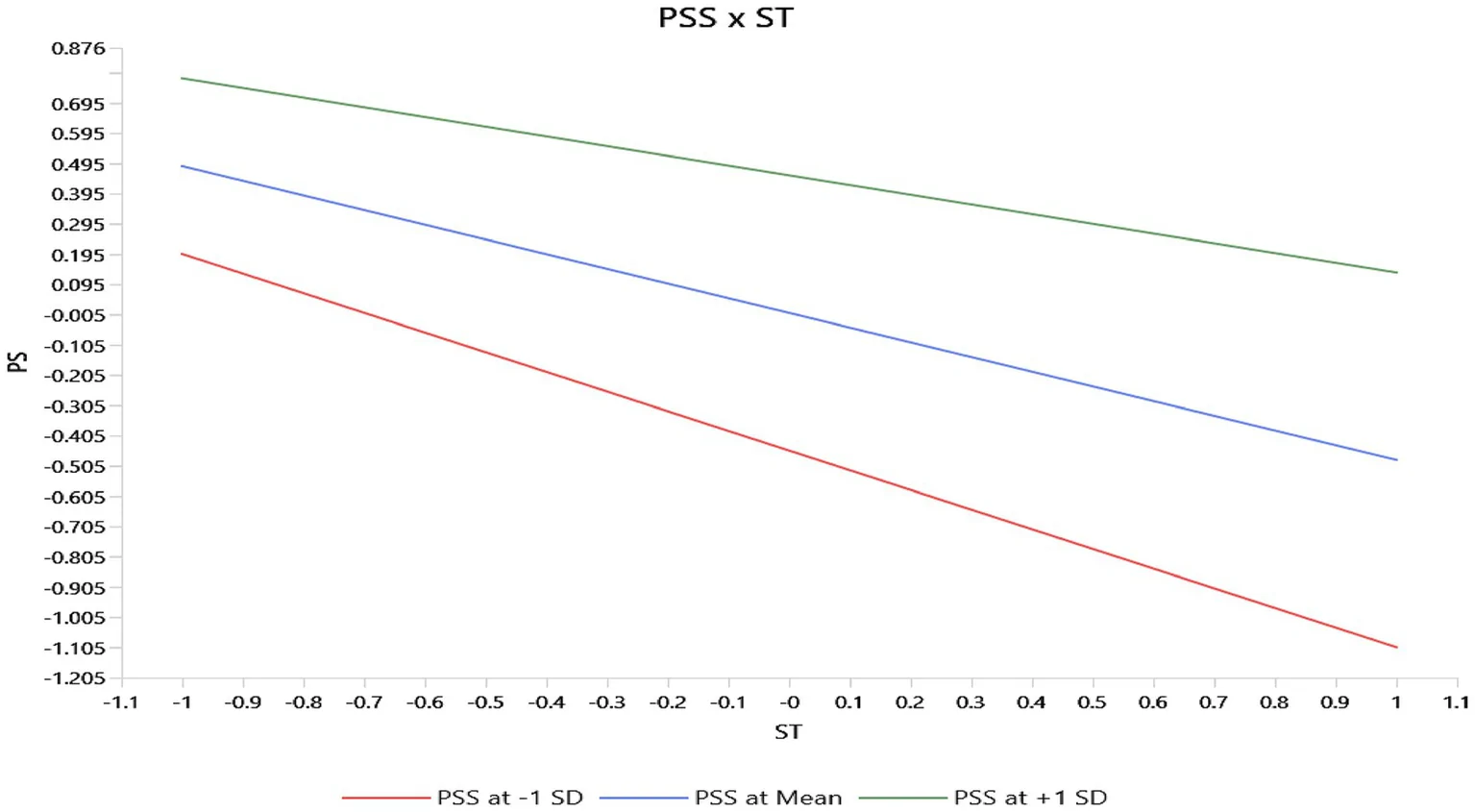
Perceived supervisor support moderates the relationship between silo thinking and psychological safety (PSS × ST → PS).

**Figure 7:**
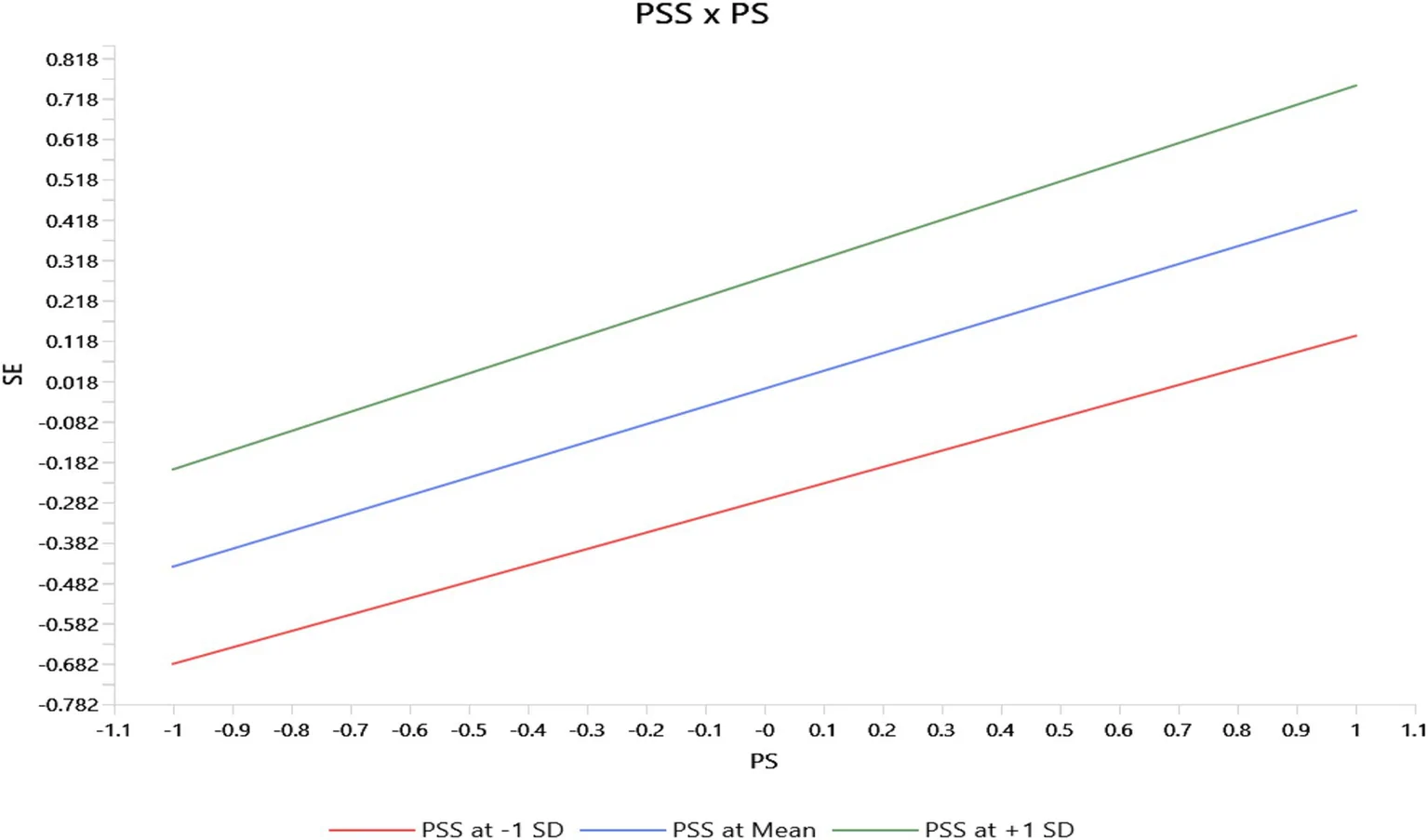
Interaction between perceived supervisor support and psychological safety in relation to staff engagement (PSS × PS → SE).

Consistent with H1, silo thinking was significantly and negatively associated with staff engagement (*β* = −0.219, *t* = 4.802, *p* < 0.001, BCa bootstrap limits [−0.293, −0.145]). This indicates that respondents reporting higher levels of silo thinking also tended to report lower levels of staff engagement. Similarly, H2 was supported, showing that silo thinking was significantly and negatively associated with psychological safety (*β* = −0.486, *t* = 14.178, *p* < 0.001, BCa bootstrap limits [−0.541, −0.430]). The larger coefficient indicates that silo thinking was more strongly negatively associated with psychological safety than with staff engagement.

As predicted in H3, psychological safety was significantly and positively associated with staff engagement (*β* = 0.441, *t* = 8.908, *p* < 0.001, BCa bootstrap limits [0.358, 0.521]). Similarly, H4 was supported, indicating that perceived supervisor support was positively associated with staff engagement (*β* = 0.275, *t* = 6.697, *p* < 0.001, BCa bootstrap limits [0.207, 0.344]). These findings indicate that respondents reporting higher levels of psychological safety and perceived supervisor support also tended to report higher levels of staff engagement.

The structural model included the direct association between perceived supervisor support and psychological safety, along with the hypothesised interaction effect between perceived supervisor support and silo thinking. This association was positive and statistically significant (*β* = 0.455, *t* = 12.319, *p* < 0.001, BCa bootstrap limits [0.395, 0.516]).

The mediation analysis supported H5. Psychological safety significantly mediated the association between silo thinking and staff engagement (*β* = −0.214, *t* = 7.238, *p* < 0.001, BCa bootstrap limits [−0.264, −0.166]). Because the bootstrap limits were entirely below zero, the indirect association was statistically significant, indicating that psychological safety partly accounts for the negative association between silo thinking and staff engagement.

The moderation analyses produced mixed findings. H6 was supported, indicating that perceived supervisor support significantly moderated the association between silo thinking and staff engagement (*β* = 0.103, *t* = 2.529, *p* = 0.006, BCa bootstrap limits [0.036, 0.170]). As illustrated in Figure 5, the simple-slope analysis shows that the negative association between silo thinking and staff engagement was strongest when perceived supervisor support was low (−1 SD) and became progressively weaker at average and high (+1 SD) levels of perceived supervisor support. Thus, respondents reporting higher levels of supervisor support exhibited a weaker negative association between silo thinking and staff engagement than those reporting lower levels of supervisor support.

Similarly, H7 was supported. Perceived supervisor support significantly moderated the association between silo thinking and psychological safety (*β* = 0.165, *t* = 4.640, *p* < 0.001, BCa bootstrap limits [0.103, 0.221]). As shown in Figure 6, the negative association between silo thinking and psychological safety was more pronounced at low levels of perceived supervisor support and became weaker as supervisor support increased. Respondents reporting higher levels of perceived supervisor support also reported higher levels of psychological safety across different levels of silo thinking.

In contrast, H8 was not supported. The interaction between perceived supervisor support and psychological safety was not statistically significant (*β* = 0.034, *t* = 0.844, *p* = 0.199, BCa bootstrap limits [−0.031, 0.103]). Figure 7 illustrates the association between psychological safety and staff engagement at low, average, and high levels of perceived supervisor support. However, the non-significant interaction term and the bootstrap limits that include zero provide no evidence that the association between psychological safety and staff engagement differed significantly across levels of perceived supervisor support.

Regarding the control variables (see Appendix F), educational attainment (EL) was positively associated with staff engagement (*β* = 0.096, *t* = 2.777, *p* = 0.003, BCa bootstrap limits [0.041, 0.154]). Respondents with higher educational qualifications reported higher levels of staff engagement. Sex was not significantly associated with staff engagement (*β* = 0.051, *t* = 0.729, *p* = 0.233, BCa bootstrap limits [−0.063, 0.170]), indicating that there was no statistically significant association between sex and staff engagement in the sample.

Overall, the findings indicate that silo thinking was negatively associated with psychological safety and staff engagement. In contrast, psychological safety partly accounted for the negative association between silo thinking and staff engagement. Perceived supervisor support moderated the associations of silo thinking with staff engagement and psychological safety, but did not significantly moderate the association between psychological safety and staff engagement.

## 5 Discussion and conclusion

### 5.1 Direct relationships (H1–H4)

This study revealed that silo thinking was significantly and negatively associated with staff engagement and psychological safety. In contrast, psychological safety and perceived supervisor support were significantly and positively associated with staff engagement. These findings collectively support the complementary perspectives of Social Capital Theory and Social Exchange Theory, indicating that organisational relationships, collaborative interactions, and reciprocal exchanges are associated with employees’ experiences in Ghana’s healthcare sector.

Consistent with H1, silo thinking was negatively associated with staff engagement. These findings indicate that individuals working in healthcare organisations characterised by rigid departmental boundaries, fragmented communication, and limited cross-functional collaboration tend to report lower levels of engagement. This finding aligns with earlier research connecting organisational fragmentation to lower employee engagement (Bento et al., 2020; Jones et al., 2024; Nurmi and Hinds, 2020). Similarly, Krishnan and Rathakrishnan (2025) and Sweetman et al. (2023) highlighted the significance of collaborative workplace relationships in maintaining staff engagement. This research expands upon existing literature by showing that these associations are also evident within Ghana’s healthcare sector, reinforcing the Social Capital Theory assertion that fragmented organisational frameworks are associated with weaker relational networks and lower staff engagement. Lah (2025) contended that fragmented systems hinder collaborative learning and organisational innovation.

The results indicated that silo thinking was inversely related to psychological safety, supporting H2. Individuals reporting higher levels of organisational fragmentation tend to report lower psychological safety. These findings align with Ahmad (2025), Carmeli and Gittell (2009), and Dodds et al. (2025), who found that fragmented organisational settings hinder communication, reduce interpersonal trust, and impede collaborative learning. Edmondson (2018) and Newman et al. (2017) posited that psychological safety emerges from nurturing interpersonal connections defined by trust and transparency. This study’s results indicate that organisational fragmentation is associated not only with operational challenges but also with lower levels of psychological safety, aligning with Social Capital Theory.

Consistent with H3, psychological safety showed a significant positive association with staff engagement. Individuals reporting higher levels of psychological safety also tended to report higher levels of work engagement. This finding aligns with the work of Kahn (1990), Ge (2020), Kyambade et al. (2024), Fait et al. (2023), Kumar (2024), Bakker and Albrecht (2018), and O'Donovan and McAuliffe (2020), all of whom identified positive correlations between psychologically safe workplaces and employee engagement. Social Capital Theory explains these relationships through trusting and collaborative workplace dynamics, whereas Social Exchange Theory suggests that employees are likely to reciprocate psychologically safe workplace experiences through greater engagement.

Furthermore, H4 demonstrated that perceived supervisor support was significantly and positively associated with staff engagement. Workers who perceive greater support from their supervisors are likely to report higher levels of work engagement. These findings align with the findings of Tan et al. (2023), Eisenberger et al. (2002), Yang et al. (2024), Campbell (2026), Tenakwah et al. (2024), Panda et al. (2022), Bakker and Demerouti (2017), and Six and Sorge (2008), all of whom have consistently documented a positive relationship between supervisory support and staff engagement. This study most robustly endorses Social Exchange Theory, indicating that employees who perceive a supportive supervisory relationship are more inclined to reciprocate with positive work attitudes. Social Capital Theory strengthens this view by emphasising the significance of supervisory relationships in fostering trust and cooperation within healthcare institutions.

### 5.2 Mediating role of psychological safety (H5)

The results additionally indicated that psychological safety partially mediated the negative relationship between silo thinking and staff engagement, supporting H5. This indicates that the negative association between silo thinking and staff engagement operates, in part, through employees’ perceptions of psychological safety. Staff members reporting higher levels of silo thinking also tended to report lower levels of psychological safety, which was associated with lower staff engagement.

These findings align with earlier research emphasising psychological safety as a crucial relational factor connecting working environments to employee outcomes (Edmondson, 2018; Newman et al., 2017; Ge, 2020). Similarly, Lewis et al. (2010) and Hamm et al. (2022) posited that fragmented organisational frameworks undermine collaborative dynamics. In contrast, O'Donovan and McAuliffe (2020) and Bakker and Albrecht (2018) indicated that psychologically safe environments promote staff engagement and knowledge sharing. This research expands on existing literature by showing that psychological safety is a crucial pathway linking silo thinking to staff engagement in Ghana’s healthcare sector.

The partial mediation result further supports the complementary premises of Social Capital Theory and Social Exchange Theory. Social Capital Theory proposes that fragmented organisational frameworks reduce interpersonal trust and collaborative interactions, consequently reducing employees’ perceptions of psychological safety. Social Exchange Theory builds on this by suggesting that employees who perceive lower levels of trust and support may be less inclined to reciprocate with favourable workplace behaviours like engagement. Collectively, these results indicate that psychological safety functions as a crucial relational mechanism connecting organisational fragmentation to staff engagement.

### 5.3 Moderating role of perceived supervisor support (H6–H7)

The results also indicated that perceived supervisor support significantly moderated the adverse relationship between silo thinking and staff engagement (H6) and between silo thinking and psychological safety (H7). The simple-slope analysis revealed that these adverse relationships diminished progressively as perceived supervisor support increased. Staff reporting higher levels of supervisor support consistently showed higher levels of psychological safety and staff engagement across varying levels of silo thinking.

These results align with other research indicating that positive supervisory relationships are associated with enhanced employee attitudes and behaviours (Tan et al., 2023; Eisenberger et al., 2002; Yang et al., 2024). In a similar vein, Bakker and Demerouti (2017), Panda et al. (2022), Campbell (2026), Tenakwah et al. (2024), and Six and Sorge (2008) underscored that supervisory support is a crucial workplace resource that enhances trust, motivation, and employees’ capacity to manage challenging organisational conditions. This research expands upon existing literature by proposing that perceived supervisor support is associated not only with increased staff engagement but also with weaker negative relationships between silo thinking and both psychological safety and staff engagement in Ghana’s healthcare sector.

These moderation results predominantly align with Social Exchange Theory, which argues that staff tend to reciprocate supportive supervisory relationships with positive workplace attitudes and behaviours (Blau, 1964). Social Capital Theory enhances this explanation by proposing that encouraging supervisors foster trust, communication, and collaboration, thus strengthening the relational resources available to employees in fragmented organisational settings. The results indicate that positive supervisory relationships are associated with weaker negative associations between silo thinking and significant employee outcomes. However, they do not eliminate the broader organisational issues associated with silo thinking.

### 5.4 Non-significant moderating effect (H8)

In contrast to H6 and H7, H8 was not supported. The interaction between perceived supervisor support and psychological safety was not statistically significant, indicating that the study found insufficient evidence that the association between psychological safety and staff engagement differed across levels of perceived supervisor support. Although Figure 7 illustrates the positive association between psychological safety and staff engagement at low, moderate, and high levels of perceived supervisor support, the non-significant interaction does not provide sufficient evidence that the strength of this association varies significantly across these levels.

This finding is consistent with previous research highlighting psychological safety as an important workplace condition associated with staff engagement (Edmondson, 2018; Newman et al., 2017; O'Donovan and McAuliffe, 2020; Bakker and Albrecht, 2018). However, the present findings do not provide sufficient evidence that perceived supervisor support significantly moderates the association between psychological safety and staff engagement.

From a theoretical perspective, the finding provides a more nuanced understanding of the roles of Social Capital Theory and Social Exchange Theory in explaining the relationships examined in this study. Social Exchange Theory suggests that supportive supervisory relationships are associated with favourable employee attitudes through reciprocal exchanges (Blau, 1964). However, the non-significant interaction observed in H8 does not provide sufficient evidence that perceived supervisor support moderates the association between psychological safety and staff engagement. Similarly, although Social Capital Theory positions psychological safety as an important relational resource within organisations, the present findings do not establish that the strength of its association with staff engagement varies according to perceived supervisor support.

## 6 Theoretical contributions

This study contributes to the literature by extending understanding of the relational processes associated with staff engagement within healthcare organisations. The results indicate that integrating Social Capital Theory and Social Exchange Theory helps explain how organisational frameworks and interpersonal connections collaboratively shape employees’ experiences at work. Rather than analysing these theoretical frameworks in isolation, the research highlights their complementary roles in explaining the relationship between organisational fragmentation, psychological safety, and supervisory support in relation to staff engagement.

This research indicates that silo thinking is negatively associated with staff engagement within organisations. In contrast to studies that mainly emphasise collaboration and support (Kahn, 1990; Bakker and Demerouti, 2008), the results suggest that organisational fragmentation is associated with lower staff engagement, potentially because it impairs relational ties, trust, mutual understanding, and interdepartmental communication.

This study also advances Social Capital Theory by conceptualising and empirically supporting silo thinking as a multidimensional higher-order construct comprising values, the collaborative operating model, the collaborative environment, leadership, and people reward and development. This conceptualisation extends existing research by suggesting that silo thinking reflects a broader relational phenomenon rather than a single organisational characteristic. This study further advances Social Capital Theory by suggesting that silo thinking may be understood as a phenomenon that reduces relational resources rather than merely a coordination issue (Nahapiet and Ghoshal, 1998; Zhai et al., 2025). Contemporary studies on social capital emphasise the advantages of networks, reciprocity, and trust. The present findings, however, suggest that structural fragmentation is associated with weaker relational assets and lower staff engagement. This perspective broadens engagement research by highlighting that the absence of relational capital may be as important as its presence in understanding staff engagement.

This research strengthens earlier investigations into psychological safety by proposing that it serves as a crucial relational mechanism connecting silo thinking with staff engagement, reinforcing previous findings that highlight its relationship with learning, communication, and a sense of belonging (McClintock and Fainstad, 2022). The mediation results show that psychological safety partially accounts for the association between silo thinking and staff engagement, while also implying that other organisational factors may play a role in this relationship. Together, these results enhance relational theories by emphasising the integrated influence of organisational fragmentation, interpersonal relationships, and psychological safety on staff engagement.

The results enhance Social Exchange Theory by revealing that perceived supervisor support acts as a contextual boundary condition rather than merely a direct organisational resource. Supportive supervision is frequently considered a significant element linked to staff engagement (Bachkirova et al., 2021). This study’s results indicate that supervisory support is especially significant in weakening the negative associations between silo thinking and both psychological safety and staff engagement, rather than enhancing all workplace interactions equally. These findings extend Social Exchange Theory by suggesting that mutual supervisor–employee exchanges may become more important in contexts of organisational fragmentation.

The non-significant moderating role of perceived supervisor support in the relationship between psychological safety and staff engagement provides an additional theoretical insight. The study found insufficient evidence that the association between psychological safety and staff engagement differed across levels of perceived supervisor support. This finding suggests that the relationship between psychological safety and staff engagement should be interpreted without assuming a significant moderating role for perceived supervisor support in the present sample. Consequently, the findings contribute to Social Exchange Theory and Social Capital Theory by highlighting the importance of considering the contextual conditions under which relational resources operate, while acknowledging that perceived supervisor support did not significantly moderate the relationship between psychological safety and staff engagement.

Finally, these results broaden theoretical views on the “dark side” of social capital by indicating that silo thinking could diminish the relational and cognitive aspects of social capital. Rather than viewing organisational structures merely as mechanisms for collaboration, the results suggest that structural fragmentation may hinder trust, inclusivity, and collaborative relationships. Collectively, the study contributes to a more integrated theoretical framework in which staff engagement is understood as being associated with organisational structures, relational climates, and supervisory relationships operating simultaneously within complex organisational systems.

## 7 Practical implications

The results of this research provide significant practical guidance for hospital administrators, managers, and policymakers. The identified relationships suggest that both formal organisational frameworks and the quality of relational practices are associated with staff engagement in healthcare organisations. In particular, the negative association between silo thinking and staff engagement suggests that hospitals and other healthcare organisations may benefit from moving beyond rigid departmental boundaries by fostering more interconnected work systems. Administrators might consider formalising interdepartmental forums, collaborative clinical meetings, and cross-functional task forces as routine organisational practices. Such endeavours facilitate communication, build trust, and support collaborative problem-solving. Similarly, integrated care pathways and periodic collaboration across organisational units may strengthen interpersonal familiarity and working relationships among different professional groups.

This study did not empirically assess the effectiveness of these organisational strategies; instead, the suggestions are based on observed statistical correlations rather than established causal relationships. Accordingly, they should be interpreted as evidence-informed considerations that may help healthcare organisations foster organisational conditions associated with stronger staff engagement, rather than as interventions that will necessarily produce these outcomes.

From an operational standpoint, addressing silo thinking may necessitate a focus on both relational and structural dynamics. Creating consistent communication pathways among healthcare professionals across different departments and disciplines could enhance relational coordination, reduce feelings of isolation, and foster a collective sense of purpose within organisations. These organisational practices also create conditions that support collaboration, informed decision-making, and coordinated patient care. In healthcare settings characterised by high interdependence, relational integration serves as a crucial managerial strategy for enhancing staff morale, organisational effectiveness, and service quality.

The results also imply that health authorities should consider integrating relational and engagement-focused indicators into national health policies and regulatory structures. Accreditation and performance assessment frameworks may incorporate metrics of interdepartmental collaboration, psychological safety, and staff engagement to motivate healthcare organisations to strengthen these organisational practices. Although the current research does not determine causal relationships, the results suggest that these relational aspects deserve further attention in healthcare policy and organisational assessment. Policies that promote engagement, support, and shared responsibility could strengthen healthcare systems’ resilience.

The research additionally provides actionable recommendations for managers. The results indicate that perceived supervisor support is a significant relational asset linked to staff engagement and psychological safety. Supervisors can enhance workplace conditions by providing regular feedback, acknowledging employee efforts, fostering teamwork, and maintaining safe opportunities for employees to voice concerns. The findings further suggest that effective supervisory support extends beyond formal procedures and may be strengthened through regular communication, trust-building, genuine concern for employees, and inclusive leadership practices.

The non-significant moderating effect of perceived supervisor support on the association between psychological safety and staff engagement suggests that supervisors should not rely exclusively on supervisory support to sustain employee engagement. Supportive supervision is likely most effective when integrated with broader organisational initiatives that enhance collaboration, trust, and psychologically safe work settings.

Finally, the results indicate that healthcare institutions should enhance workplace dynamics by addressing silo thinking while simultaneously fostering supervisory strategies that promote collaboration and employee support. Such measures could enhance staff retention, organisational robustness, and workforce sustainability. Although this research cannot definitively prove that these interventions will enhance staff engagement, the results suggest that they may be important organisational practices deserving of attention in resource-limited healthcare environments and may align with broader healthcare sustainability objectives, such as the Sustainable Development Goals (SDGs).

## 8 Limitations and directions for future studies

Like other empirical studies, this study has limitations that should be considered when interpreting its findings. Using a self-reported survey can introduce common method variance and social desirability bias, as participants’ responses may reflect perceptions rather than objective assessments of workplace experiences. The study used Harman’s single-factor test and the full-collinearity test to assess this concern; however, reliance on self-reported data remains an inherent limitation. Future studies could strengthen the evidence base by combining self-reported measures with supervisor assessments, peer evaluations, or objective organisational indicators.

The study employed a purposive, multi-stage sampling approach rather than probability sampling. Although this approach enabled the inclusion of diverse public and private hospitals and a broad range of healthcare professionals, the findings should not be interpreted as statistically representative of all healthcare employees or healthcare institutions in Ghana. Future studies may employ probability-based sampling techniques to enhance the generalisability of the findings.

Hospital administrators and human resource managers facilitated questionnaire distribution as institutional gatekeepers. Although this approach improved organisational access and enabled the survey to reach eligible participants across multiple departments, institutional representatives may have influenced participation decisions or response behaviour despite the confidentiality assurances provided. Future studies may consider combining organisational access with independent recruitment procedures where feasible.

The study’s cross-sectional design also limits the ability to establish definitive causal relationships among the variables. Although theory and statistical modelling supported the proposed relationships, the findings should be interpreted as statistical associations rather than causal effects. Future research could adopt longitudinal or experimental designs to examine how silo thinking, perceived supervisor support, psychological safety, and staff engagement evolve.

This study included participants from both public and private hospitals; however, it did not compare hospital types. Organisational differences in structure, resource availability, leadership practices, and workplace culture may be associated with variations in the observed relationships. Future studies could undertake comparative analyses across different categories of healthcare institutions to determine whether the relationships identified in this study remain consistent across diverse organisational contexts.

Another limitation is the potential clustering of observations within healthcare institutions. Although hospitals were not treated as the units of analysis, the 363 healthcare professionals were drawn from 25 healthcare institutions and may have shared organisational conditions, supervisory practices, and workplace dynamics. The available information did not allow assessment of the intraclass correlation or the extent of between-institution clustering. Consequently, the present analysis did not explicitly account for the data’s nested structure. Future research could address this issue by collecting institution-level information and applying multilevel modelling or cluster-robust standard errors to account for potential dependence among observations within healthcare institutions.

Furthermore, the instruments were not pilot-tested or subjected to expert content evaluation before the main survey. Although the measures were drawn from previously validated scales, the absence of additional contextual validation may have limited the assessment of their clarity and contextual relevance among Ghanaian healthcare professionals.

Finally, although all measurement instruments were adapted from previously validated scales and demonstrated satisfactory psychometric properties, the adaptation process may have introduced contextual variations specific to the Ghanaian healthcare setting. Future studies should further validate these adapted instruments across different healthcare settings and cultural contexts to strengthen their generalisability and replicability.

## 9 Conclusion

This study examined the associations among silo thinking, psychological safety, perceived supervisor support, and staff engagement in Ghana’s healthcare sector. The findings suggest that silo thinking was negatively associated with psychological safety and staff engagement, whereas psychological safety and perceived supervisor support were positively associated with staff engagement. Psychological safety partially mediated the relationship between silo thinking and staff engagement, while perceived supervisor support weakened the negative associations between silo thinking and both psychological safety and staff engagement. However, perceived supervisor support did not significantly moderate the relationship between psychological safety and staff engagement. Overall, the findings extend the complementary perspectives of Social Capital Theory and Social Exchange Theory by highlighting the importance of organisational relationships and supportive supervisory practices in understanding staff engagement within healthcare organisations.

## Data Availability

The original contributions presented in the study are included in the article/Supplementary material, further inquiries can be directed to the corresponding authors.
