# Supplementary material for: Breaking down silo thinking: the role of perceived supervisor support, psychological safety, and staff engagement in Ghana’s healthcare sector
Source: Front Psychol. 2026 Oct 20;17:1921650. doi: 10.3389/fpsyg.2026.1921650 (PMC13727464; doi:10.3389/fpsyg.2026.1921650)
Supplement: Supplementary file 1 [file Supplementary_file_1.docx]

**Appendix A: Sample Size Calculation Based on G*Power**

**Appendix B**: Questionnaire

| **Construct** | **Dimension/Description** | **Source** |
| --- | --- | --- |
| Silo thinking | **Values** | de Waal et al. (2019) |
|  | Create and enforce a set of values that support collaboration |  |
|  | Promote a shared identity that brings organisational units together |  |
|  | Focus on creating a united collaborative mind-set |  |
|  | Create clear unifying goals for people to work on together |  |
|  | **Collaborative operating model** |  |
|  | Create clarity of roles, responsibilities, goals, tasks, and outcomes |  |
|  | Standardise processes, procedures, and roles across organisational units to foster collaboration |  |
|  | Integrate process and systems across organisational units |  |
|  | Develop interdependent planning and review processes across organisational units |  |
|  | Develop, by multiple organisational units, products/services for customers |  |
|  | Create cross-organisational unit programs and projects for people to stimulate collaboration |  |
|  | Allow time and space to conduct cross-organisational unit experimentation and innovation |  |
|  | Devise and implement methods for resolving cross-organisational unit conflict and disagreement and build trust |  |
|  | Develop new indicators to measure, discuss, and evaluate the quality and success of collaboration |  |
|  | Install a common information technology (IT) platform and systems across all organisational units |  |
|  | Use IT systems to enable better information sharing across organisational units |  |
|  | **Collaborative environment** |  |
|  | Ensure equality and justice in the treatment of all organisational units |  |
|  | Create open information flows to share information on goals, plans, and results throughout the organisation |  |
|  | Inform organisational units about the goals and status of other organisational units |  |
|  | Create communities/networks to share knowledge, practice, and experience across organisational units |  |
|  | Hold cross-organisational unit training and events to build inter-organisational unit respect, understanding, and trust |  |
|  | Create a physical space where colleagues can collide and bond |  |
|  | Encourage colleagues to spend time with colleagues from other organisational units |  |
|  | Create informal settings for people to get to know each other |  |
|  | **Leadership** |  |
|  | Managers have responsibility for results in their own area and share responsibilities elsewhere |  |
|  | Ensure senior leadership demonstrates collaborative behaviour |  |
|  | Develop interpersonal skillsets within managers to better enable them to collaborate and network |  |
|  | **People reward and development** |  |
|  | Recruit people with a collaborative mind-set and good networking skills |  |
|  | Provide employees with training to further develop their collaborative and networking skills |  |
|  | Give people the authority and accountability to act in a collaborative manner |  |
|  | Adapt evaluation, reward, and incentive systems to specifically reward cross-organisational unit collaborative efforts and results |  |
|  | Visibly recognise and reward people who collaborate across organisational units |  |
| Psychological safety | I am able to bring up problems and tough issues. | Edmondson (1999) |
|  | People in this organisation do not reject others for being different. |  |
|  | It is safe to take a risk in this organisation. |  |
|  | It is easy for me to ask other members of the organisation for help. |  |
|  | No one in this organisation would deliberately act in a way that undermines my efforts. |  |
| Staff engagement | **Absorption** | Schaufeli and Bakker (2004) |
|  | I feel happy when I am working intensely |  |
|  | I am immersed in my work |  |
|  | It is difficult to detach myself from my job |  |
|  | **Dedication** |  |
|  | I am enthusiastic about my job |  |
|  | My job inspires me |  |
|  | To me, my job is challenging |  |
|  | **Vigour** |  |
|  | At my work, I feel bursting with energy |  |
|  | At my job, I am very resilient, mentally |  |
|  | At my work I always persevere, even when things do not go well |  |
| Perceived supervisor support | My supervisor cares about my opinions | Siddiqi et al. (2025) |
|  | My supervisor cares about my well-being |  |
|  | My supervisor strongly considers my goals and values |  |
|  | My supervisor is willing to help me when I need a special favour |  |
|  | My supervisor shows very little concern for me. (R) |  |

**Appendix C**: Common Method Bias (Harman’s single-factor test)

| **Total Variance Explained** | | | | | | |
| --- | --- | --- | --- | --- | --- | --- |
| Factor | Initial Eigenvalues | | | Extraction Sums of Squared Loadings | | |
|  | Total | % of Variance | Cumulative % | Total | % of Variance | Cumulative % |
| 1 | 20.733 | 41.466 | 41.466 | 20.226 | 40.451 | 40.451 |
| 2 | 6.945 | 13.890 | 55.356 |  |  |  |
| 3 | 1.810 | 3.619 | 58.975 |  |  |  |
| 4 | 1.258 | 2.517 | 61.492 |  |  |  |
| 5 | 0.848 | 1.696 | 63.187 |  |  |  |
| 6 | 0.779 | 1.557 | 64.745 |  |  |  |
| 7 | 0.744 | 1.487 | 66.232 |  |  |  |
| 8 | 0.716 | 1.431 | 67.663 |  |  |  |
| 9 | 0.687 | 1.373 | 69.037 |  |  |  |
| 10 | 0.673 | 1.345 | 70.382 |  |  |  |
| 11 | 0.662 | 1.323 | 71.705 |  |  |  |
| 12 | 0.605 | 1.210 | 72.915 |  |  |  |
| 13 | 0.591 | 1.181 | 74.097 |  |  |  |
| 14 | 0.572 | 1.144 | 75.241 |  |  |  |
| 15 | 0.546 | 1.092 | 76.333 |  |  |  |
| 16 | 0.529 | 1.057 | 77.390 |  |  |  |
| 17 | 0.521 | 1.041 | 78.431 |  |  |  |
| 18 | 0.512 | 1.023 | 79.454 |  |  |  |
| 19 | 0.501 | 1.003 | 80.457 |  |  |  |
| 20 | 0.491 | 0.982 | 81.439 |  |  |  |
| 21 | 0.477 | 0.953 | 82.392 |  |  |  |
| 22 | 0.471 | 0.943 | 83.335 |  |  |  |
| 23 | 0.463 | 0.926 | 84.261 |  |  |  |
| 24 | 0.436 | 0.873 | 85.134 |  |  |  |
| 25 | 0.429 | 0.858 | 85.992 |  |  |  |
| 26 | 0.420 | 0.841 | 86.832 |  |  |  |
| 27 | 0.409 | 0.817 | 87.649 |  |  |  |
| 28 | 0.397 | 0.793 | 88.443 |  |  |  |
| 29 | 0.381 | 0.762 | 89.205 |  |  |  |
| 30 | 0.367 | 0.734 | 89.938 |  |  |  |
| 31 | 0.349 | 0.699 | 90.637 |  |  |  |
| 32 | 0.331 | 0.662 | 91.299 |  |  |  |
| 33 | 0.322 | 0.645 | 91.944 |  |  |  |
| 34 | 0.322 | 0.643 | 92.588 |  |  |  |
| 35 | 0.311 | 0.622 | 93.210 |  |  |  |
| 36 | 0.303 | 0.606 | 93.815 |  |  |  |
| 37 | 0.287 | 0.573 | 94.389 |  |  |  |
| 38 | 0.272 | 0.544 | 94.932 |  |  |  |
| 39 | 0.260 | 0.520 | 95.453 |  |  |  |
| 40 | 0.255 | 0.511 | 95.963 |  |  |  |
| 41 | 0.244 | 0.488 | 96.451 |  |  |  |
| 42 | 0.229 | 0.458 | 96.909 |  |  |  |
| 43 | 0.222 | 0.444 | 97.353 |  |  |  |
| 44 | 0.220 | 0.441 | 97.794 |  |  |  |
| 45 | 0.207 | 0.414 | 98.208 |  |  |  |
| 46 | 0.197 | 0.394 | 98.603 |  |  |  |
| 47 | 0.186 | .372 | 98.975 |  |  |  |
| 48 | 0.177 | 0.354 | 99.329 |  |  |  |
| 49 | 0.174 | 0.349 | 99.678 |  |  |  |
| 50 | 0.161 | 0.322 | 100.000 |  |  |  |
| Extraction Method: Principal Axis Factoring. | | | | | | |

**Appendix D:** Full-Collinearity Assessment Model Using a Random Variable

**Appendix E**: Indicator Variable

**Appendix F**: Control Variable Model
